# Subunit-Specific Photocontrol of Glycine Receptors by Azobenzene-Nitrazepam Photoswitcher

**DOI:** 10.1523/ENEURO.0294-20.2020

**Published:** 2021-01-15

**Authors:** Galyna Maleeva, Alba Nin-Hill, Karin Rustler, Elena Petukhova, Daria Ponomareva, Elvira Mukhametova, Alexandre MJ Gomila, Daniel Wutz, Mercedes Alfonso-Prieto, Burkhard König, Pau Gorostiza, Piotr Bregestovski

**Affiliations:** 1 Institut National de la Santé et de la Recherche Médicale, Institut de Neurosciences des Systèmes, Aix-Marseille University, Marseille 13005, France; 2 Institute for Bioengineering of Catalonia, The Barcelona Institute of Science and Technology, Barcelona 08028, Spain; 3 Department of Inorganic and Organic Chemistry (Section of Organic Chemistry) and Institute of Theoretical Chemistry (IQTCUB), University of Barcelona, Barcelona 08028, Spain; 4 Institute of Organic Chemistry, University of Regensburg, Regensburg 93053, Germany; 5 Department of Normal Physiology, Kazan State Medical University, Kazan 420012, Russia; 6 Institute of Neurosciences, Kazan State Medical University, Kazan 420012, Russia; 7 Open Lab of Motor Neurorehabilitation, Kazan Federal University, Kazan 420008, Russia; 8 Institute for Advanced Simulation IAS-5 and Institute of Neuroscience and Medicine INM-9, Computational Biomedicine, Forschungszentrum Jülich, Jülich 52425, Germany; 9 Cécile and Oskar Vogt Institute for Brain Research, Medical Faculty, Heinrich Heine University Düsseldorf, Düsseldorf 40225, Germany; 10 Catalan Institution for Research and Advanced Studies, Barcelona 08003, Spain; 11 Centro de Investigación Biomédica en Red en Bioingeniería, Biomateriales y Nanomedicina, Madrid 28001, Spain

**Keywords:** brain slices, glycine receptors, hypoglossal motoneurons, molecular modelling, patch-clamp, photopharmacology

## Abstract

Photopharmacology is a unique approach that through a combination of photochemistry methods and advanced life science techniques allows the study and control of specific biological processes, ranging from intracellular pathways to brain circuits. Recently, a first photochromic channel blocker of anion-selective GABA_A_ receptors, the azobenzene-nitrazepam-based photochromic compound (Azo-NZ1), has been described. In the present study, using patch-clamp technique in heterologous system and in mice brain slices, site-directed mutagenesis and molecular modeling we provide evidence of the interaction of Azo-NZ1 with glycine receptors (GlyRs) and determine the molecular basis of this interaction. Glycinergic synaptic neurotransmission determines an important inhibitory drive in the vertebrate nervous system and plays a crucial role in the control of neuronal circuits in the spinal cord and brain stem. GlyRs are involved in locomotion, pain sensation, breathing, and auditory function, as well as in the development of such disorders as hyperekplexia, epilepsy, and autism. Here, we demonstrate that Azo-NZ1 blocks in a UV-dependent manner the activity of α2 GlyRs (GlyR2), while being barely active on α1 GlyRs (GlyR1). The site of Azo-NZ1 action is in the chloride-selective pore of GlyR at the 2’ position of transmembrane helix 2 and amino acids forming this site determine the difference in Azo-NZ1 blocking activity between GlyR2 and GlyR1. This subunit-specific modulation is also shown on motoneurons of brainstem slices from neonatal mice that switch during development from expressing “fetal” GlyR2 to “adult” GlyR1 receptors.

## Visual Abstract

**Figure:**
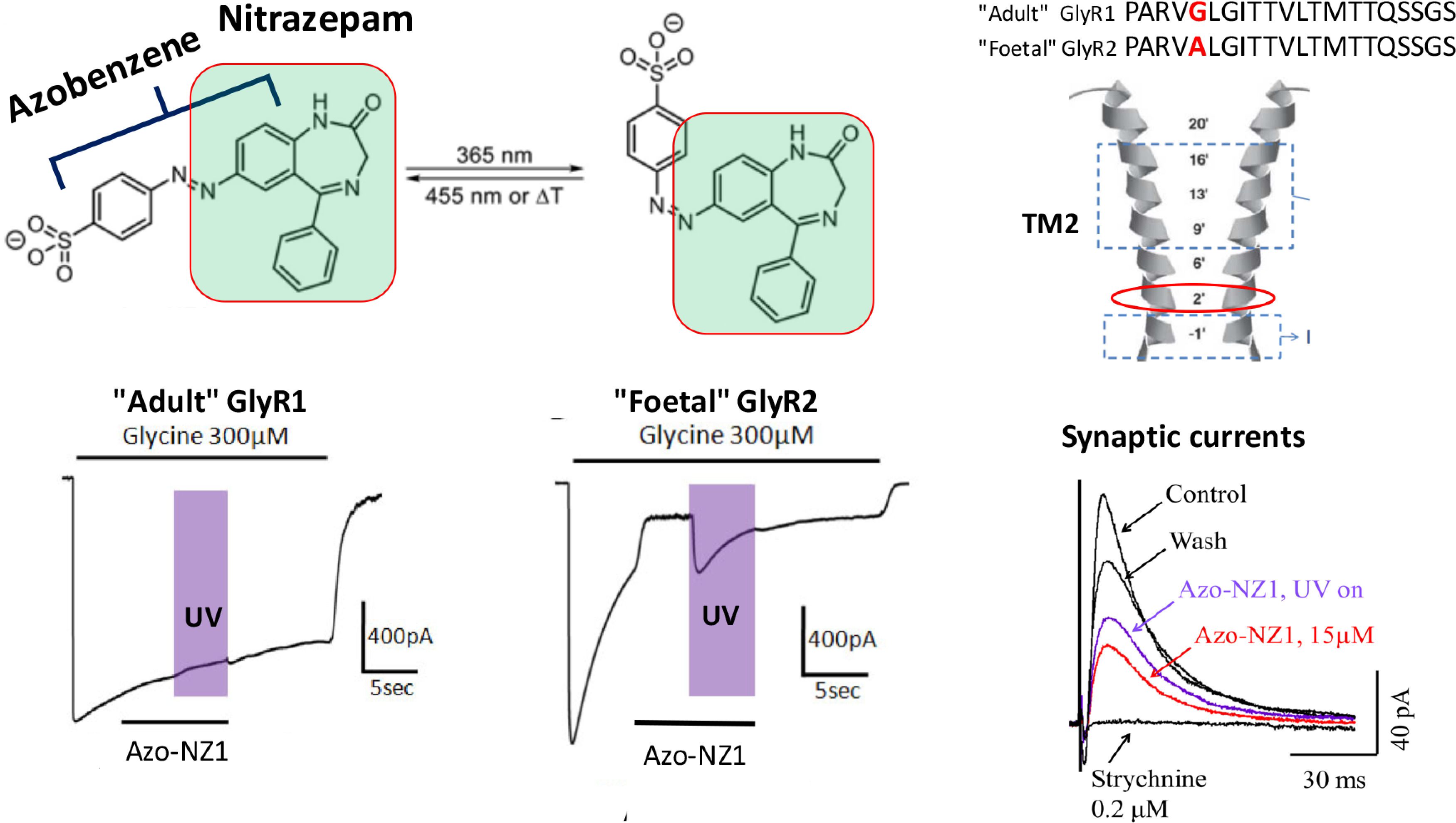

## Significance Statement

Photochromic molecules are becoming widely used for studying and modulating various biological processes. Successful application of these compounds, whose activity can be controlled with light, potentially provides a promising tool for future therapeutic approaches. The main advantage of such compounds is their precise spatial and temporal selectivity, a property that favors specific drug action and diminishes their side effects. In the present study, we describe in detail the interaction of the novel azobenzene-nitrazepam-based photochromic compound (Azo-NZ1) with glycine receptors (GlyRs) and determine its subunit-specific blocking activity in the Cl-selective pore of GlyRs. This compound offers a new strategy for specific control of glycinergic circuits and stepping stone for design of new GlyR-active drugs.

## Introduction

Photopharmacology, a rapidly developing field of research, provides a unique strategy for precise light-driven control of the activity of biological molecules (Broichhagen et al., 2015). Several classes of photochromic compounds have already been successfully used to selectively modulate the function of various proteins, including enzymes (Harvey and Abell, 2001), synaptic receptors, and voltage-gated channels (Banghart et al., 2004, 2009; Gorostiza and Isacoff, 2007, 2008; Tochitsky et al., 2012; Fehrentz et al., 2018). A number of photochromic agonists and antagonists are available for specific control of ionotropic excitatory kainate, AMPA or NMDA receptors (Volgraf et al., 2006; Kienzler et al., 2013; Berlin et al., 2016), as well as nicotinic acetylcholine receptor (Bartels et al., 1971; Lester et al., 1979; Tochitsky et al., 2012). Several optically switchable modulators of inhibitory GABA_A_ receptors were developed as well (Lin et al., 2015; Huckvale et al., 2016).

It has been recently shown that azobenzene-nitrazepam-based photochromic compound (Azo-NZ1), the azobenzene derivative of nitrazepam, blocks in a UV-dependent manner the ion channel pore of GABA_A_ α1/β2/γ2 and GABA_C_ rho2 receptors and controls GABAergic currents (Maleeva et al., 2019). These experiments also suggested that Azo-NZ1 is capable to modulate the activity of another subtype of Cys-loop receptors, α2 glycine receptor (GlyR). In the present study, we investigate in detail the profile of Azo-NZ1 action on different subtypes of GlyRs.

GlyRs are ligand-gated anion-permeable ion channels that belong to the Cys-loop receptor superfamily and provide the main inhibitory drive in the brain stem and spinal cord of vertebrates, as well as regulate network excitability in the retina, hippocampus and amygdala (Zarbin et al., 1981; McCool and Botting, 2000; Chattipakorn and McMahon, 2002; Haverkamp et al., 2003). GlyRs participate in movement control, breathing and processing of sensory information (Schmid et al., 1991; Sassoe-Pognetto et al., 1994; Harvey et al., 2004).

GlyRs represent an important drug target as they are involved in disorders such as hyperekplexia, inflammatory pain sensitization, autism, and temporal lobe epilepsy (Lynch et al., 2017; Zeilhofer et al., 2018). Strychnine is a competitive antagonist of all GlyRs subtypes and is commonly used in electrophysiological experiments to identify glycinergic IPSCs (Young and Snyder, 1973). Numerous studies have shown that strychnine and glycine binding pockets greatly overlap (Vandenberg et al., 1992; Vafa et al., 1999; Du et al., 2015). Several other compounds modulate the activity of GlyRs with strong or partial subunit selectivity. Bicuculline, a classical GABA receptor antagonist, is capable, although with lower efficacy, to also inhibit GlyRs (Fucile et al., 1999). Interestingly, it is much more effective at inhibiting GlyRα2 than GlyRα1 and hence has even been suggested to use for separation of GlyR subtypes (Wang and Slaughter, 2005). Compounds acting on GlyRs as ion channel blockers, also exhibited subunit selectivity. Cyanotriphenylborate (CTB; Rundström et al., 1994) and ginkgolide B, extracted from *Ginkgo biloba* leaves (Kondratskaya et al., 2005), have been shown as more potent blockers of GlyRα1 compared with GlyRα2, while niflumic acid (NFA), a member of the fenamate class of nonsteroidal anti-inflammatory drugs, on the contrary, was more potent at blocking GlyRα2s (Maleeva et al., 2017).

Sensitivity of GlyRs to all channel blockers strongly depended on the amino acid located at the 2’ position of the ion channel pore. Alkaloids belonging to the picrotoxin (PTX) and ginkgolide groups have more complicate patterns of interaction with GlyRs of different subunit composition, but they have in common a strong dependence on the β subunit presence (Pribilla et al., 1992; Kondratskaya et al., 2005; Hawthorne et al., 2006). Such a prominent subtype-determined modulation of GlyRs is promising feature for the development of subunit-specific modulators of GlyRs that will allow a precise control of glycinergic system.

Here, we present a detailed characterization of different GlyRs subtypes interacting with Azo-NZ1, the first photo-switchable ion channel blocker of gamma-aminobutyric acid receptors (GABARs) and GlyRs. We have demonstrated that Azo-NZ1 inhibits GlyRs in a subunit specific manner, being a potent blocker of α2 GlyRs in the *trans*-conformation, while nearly inactive in *cis*-form. Using mutagenesis analysis and molecular modeling, we have shown that the amino acid at 2’ position of the ion channel pore is crucial for Azo-NZ1 blocking action.

## Materials and Methods

### Chemistry

Synthesis and photochemical characterization of Azo-NZ1 were performed as previously reported (Maleeva et al., 2019).

### Cell culture and transfection

GlyRs of different subunit composition were heterologously expressed in cultured Chinese hamster ovary (CHO) cells obtained from the American type Tissue Culture Collection (ATCC) that were maintained as previously described (Mukhtarov et al., 2013; Maleeva et al., 2015). Transfection was performed using the Lipofectamine 3000 protocol (Life Technology). Cells were transfected with cDNA of the following GlyR subunits: α1 zebrafish, α2 zebrafish, α1 human, α1 human G254A, α1 human/β mouse, α2 mouse/β mouse (hereafter α1Z, α2Z, α1H, α1H G254A, α1H/βM, and α2M/βM, respectively). For identification of transfected cells, a cDNA of green fluorescent protein (GFP) was co-transfected with cDNA of GlyRs. Three hours after the initial exposure of cells to the cDNAs, the culture medium was replaced with fresh medium containing strychnine (1 μm), to reduce the detrimental alterations of membrane potential because of activation of overexpressed GlyRs by glycine present in culture medium. Electrophysiological recordings were conducted on fluorescent cells 24–72 h after transfection.

### Electrophysiological recordings on CHO cells

Whole-cell patch-clamp recordings were held at room temperature (20–25°C) using an EPC-9 amplifier (HEKA Elektronik). Cells were continuously superfused with external solution containing the following: 140 mm NaCl, 2 mm CaCl_2_, 2.8 mm KCl, 4 mm MgCl_2_, 20 mm HEPES, and 10 mm glucose (pH 7.4, 320–330 mOsm). Intracellular solution used for filling recording patch pipettes contained the following: 140 mm KCl, 2 mm MgCl_2_, 2 mm MgATP, and 2 mm BAPTA (tetrapotassium salt) (pH 7.3, 290 mOsm). Recording pipettes were pulled from borosilicate glass capillaries (Harvard Apparatus Ltd) and had resistances of 5–10 MΩ. Rapid replacement of solutions was provided by fast application system (SF 77A Perfusion Fast-Step, Warner), placed 40–50 μm above the recorded cell. Cells with low input resistance (<150 MΩ) and a rapid run-down (>30% with repetitive application) were excluded from analysis.

Single-channel recordings were performed in the outside-out configuration, at room temperature using an EPC-9 amplifier (HEKA Elektronik). External and internal solutions were the same as those used for whole-cell recordings.

### Electrophysiological recordings on brain slices

Experiments were performed on white laboratory ICR outbred mice of both sexes at postnatal day (P)4–P8. Use of animals was conducted in accordance with the *Guide for the Care and Use of Laboratory Animals* (NIH Publication No. 85–23, revised 1996) and European Convention for the Protection of Vertebrate Animals used for Experimental and other Scientific Purposes (Council of Europe No. 123; 1985). All animal protocols and experimental procedures were approved by the Local Ethics Committee of Kazan State Medical University (No. 10; 20.12.2016). Mice had free access to food and water and were kept under natural day length fluctuations.

For electrophysiological experiments, coronal slices containing hypoglossal nucleus were obtained. Mice were decapitated; the brainstems were removed and sliced into 350-μm-thick sections using a tissue slicer (model NVSLM1, World Precision Instruments). Sections were prepared in an ice-cold high K^+^ solution, containing the following: 120 mm K-gluconate, 10 mm HEPES‐acid, 15 mm Na-gluconate, 0.2 mm EGTA, and 4 mm NaCl (pH 7.2, 290–300 mOsm). After cutting, slices were placed for 10 min at room temperature in a choline‐based solution, containing the following: 110 mm choline chloride, 2.5 mm KCl, 1.25 mm NaH_2_PO_4_, 10 mm MgCl_2_, 0.5 mm CaCl_2_, 25 mm NaHCO_3_, 10 mm glucose, and 5 mm sodium pyruvate (pH 7.3–7.4, 290–300 mOsm). Before experiments slices were incubated for 1 h in a chamber filled with an oxygenated artificial CSF (aCSF) containing the following: 126 mm NaCl, 3.5 mm KCl, 2 mm CaCl_2_, 1.3 mm MgCl_2_, 1.2 mm NaH_2_PO_4_, 10 mm glucose, and 25 mm NaHCO_3_ (pH 7.3–7.4, 290–300 mOsm).

Glycinergic evoked IPSCs (eIPSCs) were obtained from the motor neurons of the hypoglossal nucleus, as previously described (Petukhova et al., 2018). The DS3 Constant Current Isolated Stimulator (Digitimer) and a bipolar stimulating electrode were used for the induction of reliable eIPSCs. CNQX (10 μm), APV (40 μm), and bicuculline (20 μm) were routinely added to aCSF to block the glutamatergic and GABAergic synaptic transmission. Recording was conducted at room temperature in the whole-cell configuration with the holding potential of 0 mV using the EPC‐10 patch-clamp amplifier (HEKA Elektronik). Patch electrodes were filled by the intracellular solution containing the following: 130 mm Cs-gluconate, 4 mm MgATP, 10 mm phosphocreatine, 0.3 mm GTP, 10 mm HEPES, 5 mm EGTA, and 4 mm MgCl_2_ (pH 7.3; 290 mOsm). Effects of Azo‐NZ1 on the amplitudes of glycinergic eIPSCs were examined at visible light and under illumination of aCSF by diode emitting 365 nm UV (Thorlabs).

### Data analysis and statistics

To analyze the results of patch-clamp recordings, PatchMaster (HEKA Electronic), Origin 7.5 (OriginLabs), Excel 2016 (Microsoft), and Igor Pro 6.02 (WaveMetrics) software were employed to accomplish a statistical analysis of the data and to plot the graphs. Concentration-response curves using different concentrations of glycine were fitted using a nonlinear fitting routine of the Origin 7.5 software with the Hill equation:

$$\text{I }={\text{ I}}_{\text{max}}/\left(\text{1}+{\left({\text{EC}}_{\text{5}0}/\left[\text{A}\right]\right)}^{\text{nH}}\right),$$

where *I* is the normalized current amplitude induced by the agonist at concentration [A], *I_max_* is a maximal current induced at given cell, *n_H_* is the Hill coefficient and EC_50_ is the concentrations at which a half-maximum response was induced.

To quantify the inhibitory effect of Azo-NZ1, the following equation was used:

$${\text{I}}_{\text{relative}}={\text{ I}}_{\text{2}}/{\text{I}}_{\text{1}}*\text{1}00\%,$$

where *I_1_* is the amplitude of the current before application of Azo-NZ1 (control) and *I_2_* is the amplitude of the current during the application of Azo-NZ1.

To quantify the effect of UV illumination, we used the following equation:

$${\text{I}}_{\text{relative}}={\text{ I}}_{\text{3}}/{\text{I}}_{\text{1}}*\text{1}00\%,$$

where *I_1_* is the amplitude of the current before application of Azo-NZ1 (control), *I_3_* is the amplitude of the current during the application of Azo-NZ1 on UV illumination.

Taking into the account that, in the case of α2Z, α2M/βM, and α1H G254A receptors, desensitization was present, we suggested to estimate the UV effect with respect to the washout current. However, the hump current that appeared during the washing and the potentiation effect recorded on UV illumination (see also single-channel experiments) made the correct estimation of UV effect impossible. Because of these complications and the visibly prominent UV effect, we have arbitrarily fixed it at the level of the control current.

Concentration dependences of responses to different doses of Azo-NZ1 were fitted using a nonlinear fitting routine of the Origin 7.5 software (OriginLabs) with the Hill equation:

$$\text{I}={\text{I}}_{\text{max}}/\left(\text{1}+{\left(\left[\text{inh}\right]/{\text{IC}}_{\text{5}0}\right)}^{\text{nH}}\right).$$

The effect of Azo-NZ1 at each concentration was estimated through normalization of the current amplitude with respect to the amplitude of the control current. The point corresponding to the 1000 μm was taken arbitrarily.

Data are represented as mean ± SEM. Significance of differences was assessed using paired and unpaired *t* tests, paired sample Wilcoxon signed-rank test. Differences were considered significant at *p* < 0.05.

### Single-channel analysis

Data were sampled at 10 kHz using PatchMaster (HEKA Electronic) software. Further analysis was performed with the Nest-o-Patch program.

The amplitude histograms of simple openings were fitted with the sum of Gaussian functions (for α2M GlyRs) or built based on the amplitudes of only completely resolved events (α1 and α1H G254A mutant GlyRs). Kinetic parameters have to be considered as “apparent” because of the effects of undetected shutting and opening (Colquhoun and Sigworth, 1995). The open probability (NP_o_) was calculated as the total open time of the channel during a recording divided by total time of the recording (Fucile et al., 1999).

### Drugs

Stock solution of Azo-NZ1 (10 mm) was prepared using dimethylsulfoxide (DMSO) and then diluted to the final concentration by extracellular solution. All other drugs were obtained from Hello Bio and Tocris or Sigma-Aldrich. Stock solutions were prepared using DMSO or MilliQ water and kept at −20°C.

### Modelling

#### GlyR structures

Four different GlyRs were considered (homomeric α1Z, homomeric α1Z G254A, homomeric α2H and heteromeric α2M/βM). Structures of these GlyRs were modelled in the open channel state. For homomeric α1Z GlyR, we used the cryo-EM structure with glycine bound (PDB code 3JAE) from reference (Du et al., 2015). For homomeric α1Z GlyR G254A, we used the previous structure and introduced the G254A mutation *in silico* using the molefacture tool (version 1.3) in VMD (version 1.9.2; Humphrey et al., 1996). In doing so, we assume that the G254A α1Z structure is equivalent to the α2Z structure at the level of the pore-lining M2 helices.

Homology models were generated for homomeric α2H and heteromeric α2M/βM GlyRs using SWISS-MODEL (Waterhouse et al., 2018). For the latter, being aware that the stoichiometry of heteromeric GlyRs is controversial, we decided to follow a recent study (Low et al., 2018) that proposes a 3α:2β stoichiometry with a β-α-α-β-α clockwise order. The overall sequence identity between the zebrafish α1 GlyR template and the target human α2 and mouse α2 and β GlyRs is 89%, 87%, and 55%, respectively, whereas the sequence identity when only the pore-lining residues are considered is 95%, 95%, and 38%, respectively.

#### Azo-NZ1 ligand

The initial structures of the Azo-NZ1 compound (*cis-* and *trans*- isomers) were created employing Avogadro (version 1.1.1; Hanwell et al., 2012). For each isomer, two 1,4-diazepine ring conformations, M and P, were considered. These conformations differ in orientation (below or above the plane, respectively) of C3 and the phenyl substituent of C5. For classical benzodiazepines that bind to the canonical benzodiazepine allosteric site, the M conformation is the bioactive one (i.e., the one that shows higher affinity for receptor; Richter et al., 2012). However, it is not known a priori whether Azo-NZ1 would exhibit similar conformational preferences, since GlyR does not contain a benzodiazepine binding site. Therefore, all four possible ligand structures (*cis*/M, *cis*/P, *trans*/M and *trans*/P) were considered for docking. Optimized ligand structures were taken from (Maleeva et al., 2019).

#### Docking calculations

AutoDock Vina (version 1.1.2; Trott and Olson, 2010) was employed for ligand-receptor docking. Given that experimental data indicate that Azo-NZ1 binds inside the pore, we centered our search space around the M2 helices of the pore. A flexible docking approach was used, in which both the ligand and the pore-lining residues in positions 2’, 6’, 9’, 13’, and 16’ were allowed to move. The maximum energy difference between the best and worst binding modes and the exhaustiveness were set to default values (3 and 8 kcal/mol, respectively), while the maximum number of modes was increased to 20 to increase the docking sampling. This protocol was repeated 10 times starting with different random seeds, so that a total number of 200 poses was obtained for each of the four possible conformers of Azo-NZ1. The same protocol was previously used to study the binding determinants of Azo-NZ1 in the pore of GABA_A_R and GABA_C_R (Maleeva et al., 2019).

We would like to note here that, on UV light irradiation, *cis*-Azo-NZ1 may either remain in the channel (adopting another binding conformation that unblocks the pore) or exit the pore (reaching the bath solution). The outcome will depend on the mean opening time of the channel and the dissociation rate of *cis*-Azo-NZ1. With the data at hand, we cannot distinguish between the two aforementioned possibilities. Therefore, we decided to perform docking calculations not only with *trans*-Azo-NZ1, but also with the *cis* isomer, to model the system immediately after UV irradiation. These calculations are aimed at investigating the change in the receptor-ligand binding mode right after *trans*-*cis* isomerization (both in terms of ligand position along the pore and interactions with the pore-lining residues).

In order to explore the possibility of Azo-NZ1 binding to other regions of the GlyR channel, we also performed “blind” docking calculations, in which the search space includes the whole receptor. The same docking protocol as for the calculations focused on the M2 helices of the pore was used. This strategy was recently used to uncover the putative binding site of another photochromic ligand, Glyght, to GlyRs (Gomila et al., 2020).

#### Analysis of the docking results

The docking poses of each conformer were analyzed separately. For simplicity, only the *trans*/P and *cis*/P results are discussed in the main text. The results of the corresponding M conformers are almost identical (data not shown) and, in the case of the *cis* isomer, the contribution of the M conformer is expected to be minor, as it is 3.5 kcal/mol less stable than *cis*/P. We used two different approaches to pinpoint the exact binding site of Azo-NZ1 in the M2 ion channel pore. First, we analyzed the number density of the sulfonate group of the ligand poses. Previous studies have successfully used this type of analysis to identify ligand binding sites in other ion channels (Raju et al., 2013; Maleeva et al., 2019). The underlying assumption is that regions of continuous density (or high occupancy) should represent regions of tighter binding. The number density value was computed using the Volmap plugin (Cohen et al., 2006) of VMD (Humphrey et al., 1996), as done previously (Maleeva et al., 2019). The second approach was based on the analysis of the interactions between the ligand and the receptor. Ligand binding poses were clustered using the quality threshold algorithm implemented in VMD (https://github.com/luisico/clustering) and then the representative structure of the most populated cluster(s) was analyzed using the Binana algorithm (Durrant and McCammon, 2011). In the case of the blind docking calculations, we only analyzed the sulfonate number density, as these calculations are only intended to explore the possibility of alternative binding sites responsible for secondary effects of *cis*-Azo-NZ1 on α1 GlyR, other than the main channel blocking activity. The M and P conformers of each isomer (*cis*/*trans*) were pooled together to carry out this analysis, to have more sampling. All the images of the modeling section were generated with either the UCSF Chimera package (Pettersen et al., 2004) or the VMD program (Humphrey et al., 1996).

## Results

### Effect of Azo-NZ1 on α1 GlyR

**Figure 1.**
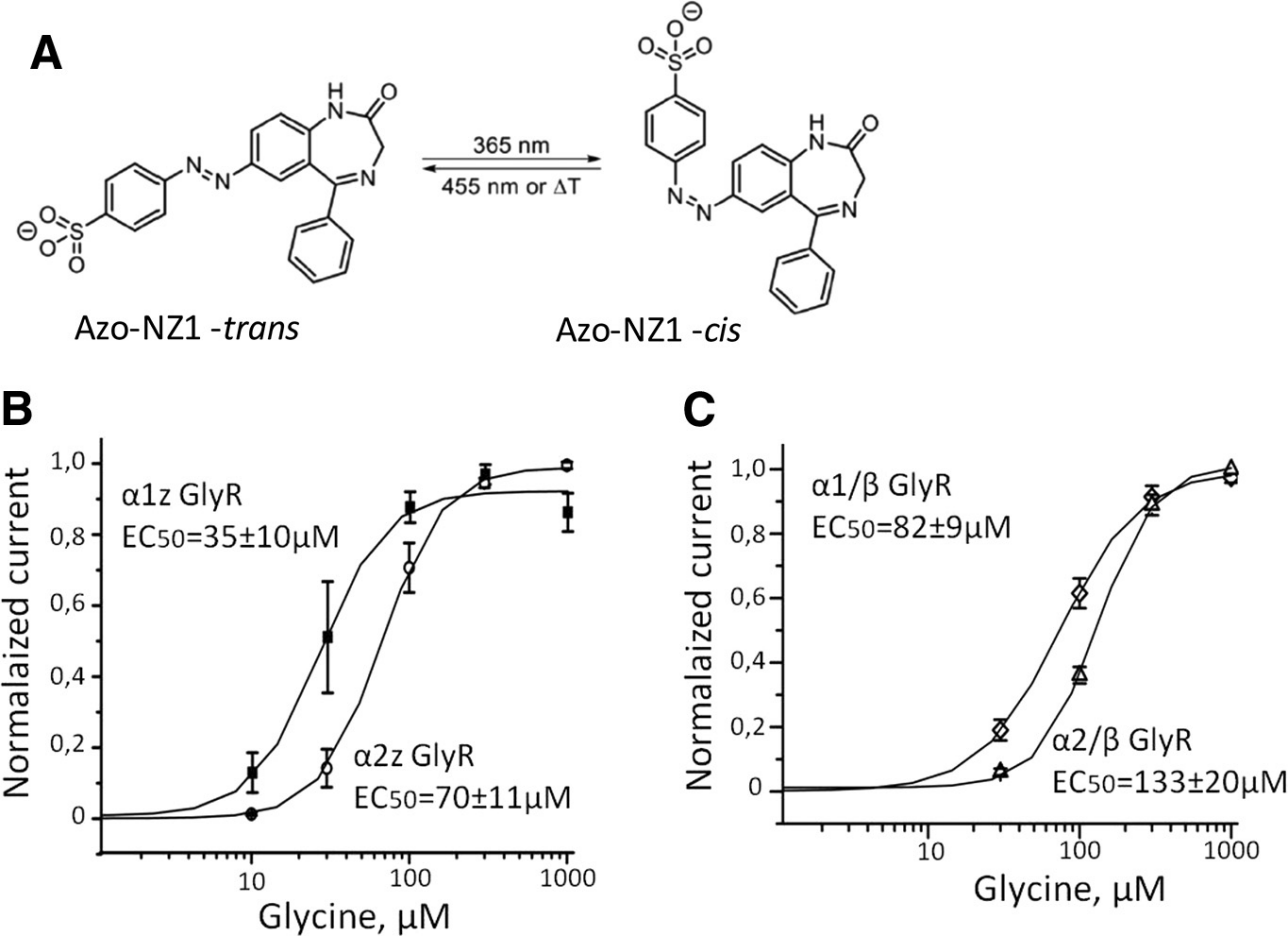
Schematic representation of Azo‐NZ1 and cumulative curves of glycine-induced dose-response dependencies for different subtypes of GlyRs. ***A***, Azo‐NZ1 in its *trans-* and *cis*‐configurations. ***B***, Cumulative dose/response curves for homomeric α1Z (filled squares, *n* = 6) and α2Z GlyRs (empty circles, *n* = 6). ***C***, Cumulative dose/response curves for heteromeric α1H/βM (diamonds, *n* = 7) and α2M/β M (triangles, *n* = 7).

Azo-NZ1 is the azobenzene derivative of nitrazepam, capable to block GABA_A_ receptor channels (Maleeva et al., 2019). Ligand pharmacological activity is regulated with the azobenzene photochromic group, which changes its configuration to the *cis* isomer when illuminated with UV-light of 365 nm. The back isomerization to the *trans* isomer can be triggered by blue or visible light (Fig. 1*A*).

To examine the effects of Azo-NZ1 on GlyRs, we first tested it at homomeric α1 zebrafish (α1Z) GlyRs expressed in CHO cells. The EC_50_ for glycine was 35 ± 10 μm (*n* = 6; Fig. 1*B*), which is similar to the previous values obtained on BOSC 23 cells transiently expressing α1Z GlyRs (David-Watine et al., 1999; Fucile et al., 1999).

**Figure 2.**
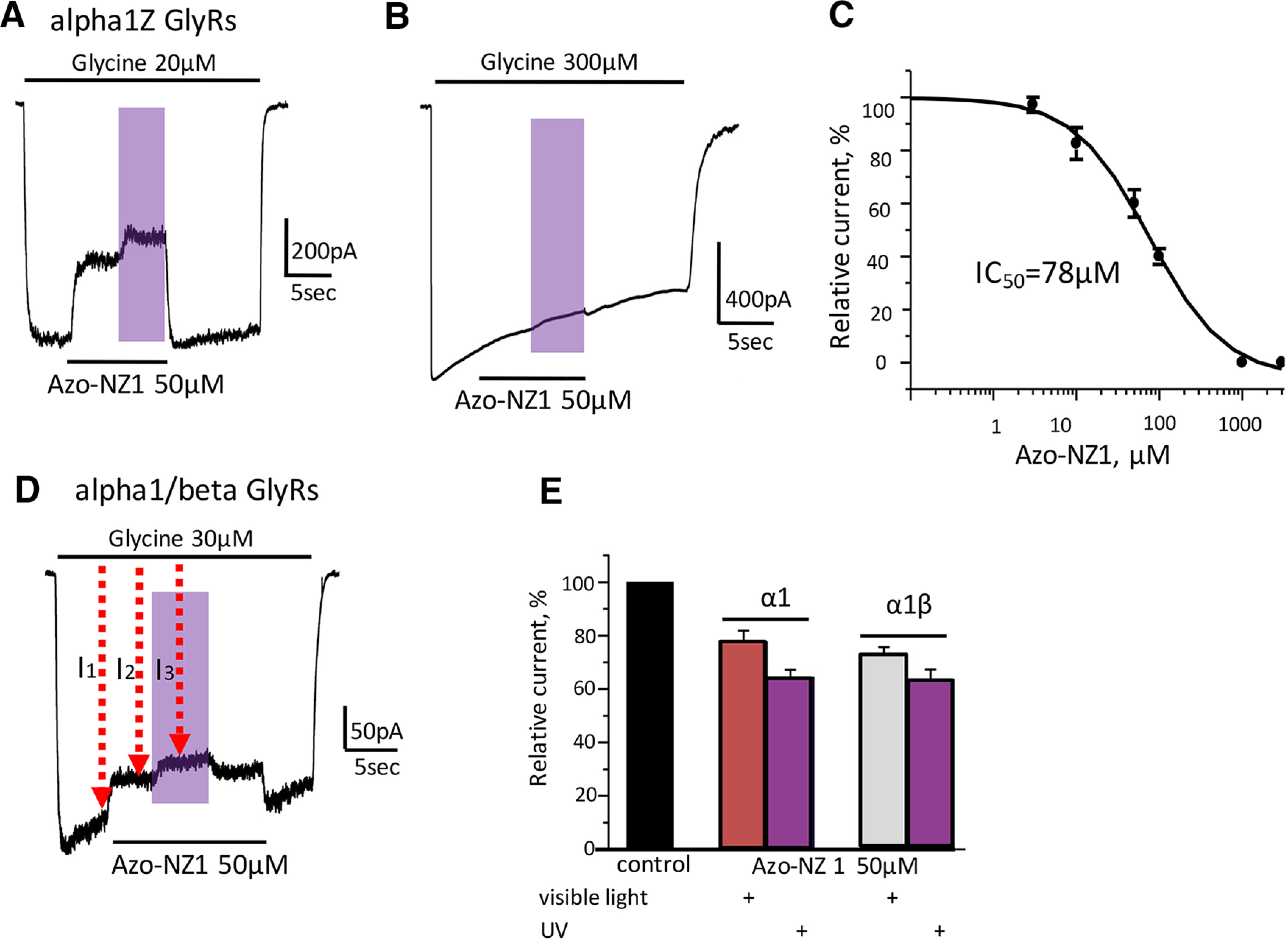
Interaction of Azo-NZ1 with homomeric α1 and heteromeric α1/β GlyRs. ***A***, Representative trace of the α1Z GlyRs-mediated current induced by application of glycine 20 μm (below EC_50_) and by mixture of glycine 20 μm with Azo-NZ1 50 μm at visible light and on illumination with UV light (here and in other figures, UV illumination is shown by violet rectangles). ***B***, Representative trace of α1Z current induced by saturating glycine concentration, 300 μm, and by a mixture of glycine 300 μm with Azo-NZ1 50 μm at visible light and on illumination with UV light. Note the absence of Azo-NZ1 inhibitory effect. ***C***, Cumulative dose–response curve for Azo-NZ1 action on α1Z GlyRs at glycine concentration of 20 μm. IC_50_ = 78 ± 8 μm, n_H_ = 1.4 ± 0.25 (*n* = 6); V_hold_ = –30 mV. ***D***, Representative trace of α1/β-mediated current induced by application of glycine 30 μm and by a mixture of glycine with Azo-NZ1 (50 μm). I_1_ and I_2_ are the amplitudes of currents used to calculate the Azo-NZ1 inhibitory effect, I_1_ and I_3_ were used to calculate the UV effect. ***E***, Relative amplitudes of α1H and α1H/βM currents induced by application of glycine 30 μm or by mixture of glycine with Azo-NZ1 50 μm. Data from three to seven cells.

Co-application of Azo-NZ1 (50 μm) with subsaturating concentration of glycine (20 μm) resulted in a decrease of current amplitude by 34 ± 2% of control (Fig. 2*A*), with an IC_50_ for Azo-NZ1 of 78 ± 8 μm and n_H_ = 1.4 ± 0.25 (*n* = 6; Fig. 2*C*). Illumination with UV light produced a slightly additional inhibition (Fig. 2*A*). Importantly, the inhibitory action of Azo-NZ1 decreased at elevation of the agonist concentration and the photochromic compound did not produce any detectable effect at α1Z GlyRs activated by saturating concentration of glycine (300 μm; Fig. 2*B*). Similar results were obtained on homomeric human GlyR receptors formed by α1 subunits, using 30 μm glycine and 50 μm Azo-NZ1 (Fig. 2*E*).

In rodents, expression of different GlyR subtypes is developmentally regulated: α2 GlyRs are predominant at birth, but during the two weeks of postnatal life α1 expression increases dramatically and becomes predominant in adult stages of life (Akagi and Miledi, 1988; Becker et al., 1988; Malosio et al., 1991). In addition, the postsynaptic clustering of GlyRs requires the β subunit (Meyer et al., 1995), and in the CNS of adult vertebrates, glycinergic synaptic transmission is predominantly provided by heteromeric α1/β receptors (Becker et al., 1988). For some compounds, the pharmacological profile of heteromeric receptors may be different from the homomeric ones (Pribilla et al., 1992). Thus, we performed analysis aimed to determine whether incorporation of β subunits will change the profile of α1 GlyRs interaction with Azo-NZ1.

Addition of Azo-NZ1 at 50 μm concentration caused inhibition of currents mediated by heteromeric α1H/βM GlyRs to 73 ± 3% with a minor strengthening of inhibition at UV illumination to 64 ± 4% (*n* = 7; Fig. 2*D*,*E*). In the same set of experiments, monomeric α1H GlyRs were inhibited by Azo-NZ1 to 78 ± 4%, with minor UV effect, to 64 ± 3% (*n* = 3; Fig. 2*E*).

These observations demonstrate that Azo-NZ1 weakly inhibits homomeric α1Z, α1H and heteromeric α1H/β GlyRs activated by low concentrations of glycine and does not block α1 GlyRs in the presence of high concentrations of the agonist.

### Effect of Azo-NZ1 on α2 GlyR

Analysis of concentration dependencies revealed that EC_50_ for glycine of α2Z GlyRs is 70 ± 11 μm (*n* = 6; Fig. 1*B*). As this value is higher than for α1 subunits, the analysis of Azo-NZ1 action on α2 GlyR was performed at glycine concentrations of 50 and 300 μm (non-saturating and saturating, respectively).

**Figure 3.**
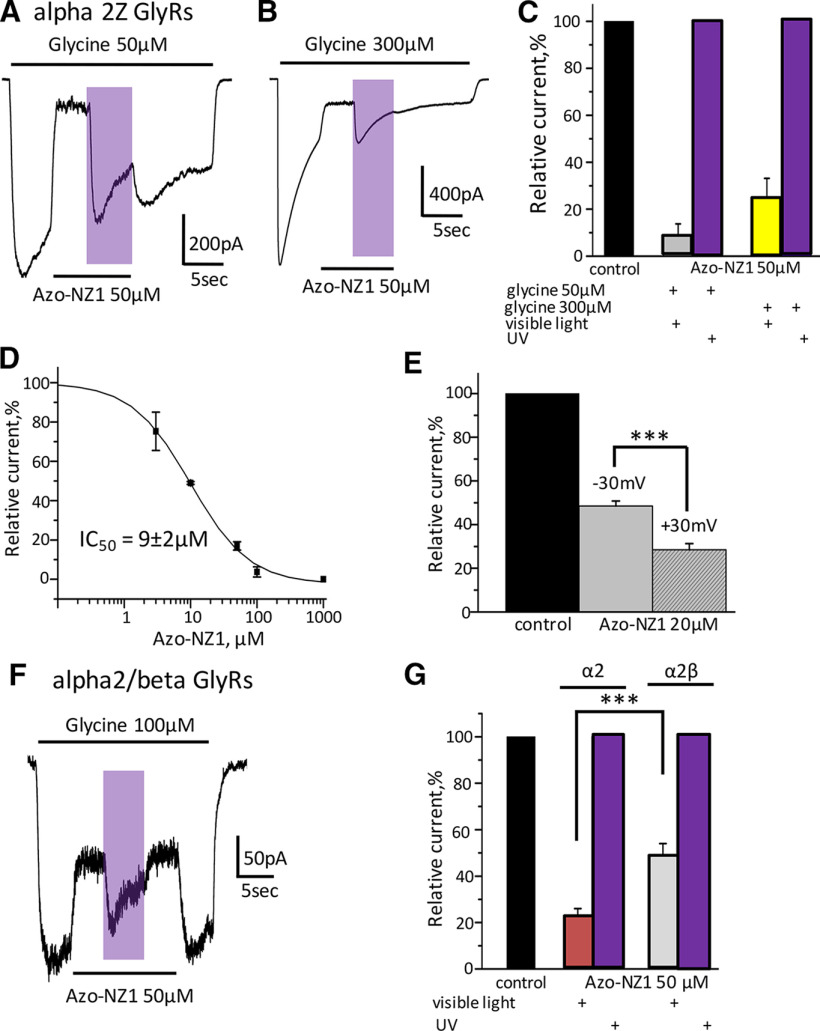
Azo-NZ1 is a potent UV-controllable inhibitor of α2 GlyRs. ***A***, Representative trace of α2Z GlyRs current induced by application of glycine 50 μm or by a mixture of glycine with Azo-NZ1 50 μm at visible light or at UV illumination. ***B***, Representative trace of α2Z current induced by application of glycine 300 μm or by a mixture of glycine with Azo-NZ1 50 μm at visible light or on UV illumination. ***C***, Cumulative data on relative amplitude of Azo-NZ1 inhibition (50 μm) of glycine-induced currents (50 and 300 μm) at visible light and on UV illumination. ***D***, Cumulative dose-response curve for Azo-NZ1; sigmoidal fitting yielded IC_50_ = 9 ± 2 μm; n_H_ = 1.02 ± 0.3 (*n* = 4); V_hold_ = –30 mV. ***E***, Cumulative data on the voltage dependence of Azo-NZ1 interaction with α2Z GlyRs (black column, relative amplitude of the current in control; gray column, on application of Azo-NZ1 20 μm at V_hold_ = –30 mV; dashed column, on application of Azo-NZ1 20 μm at V_hold_ = +30 mV); *n* = 6–8 cells, ***p ≤ 0.001. ***F***, Representative trace of α2M/βM-mediated current induced by glycine 100 μm or by mixture of glycine with Azo-NZ1 (50 μm) at visible light or on UV illumination. ***G***, Relative amplitudes of current mediated by α2M and α2M/βM receptors during the application of Azo-NZ1 50 μm at visible light and on illumination with UV light (*n* = 8–10 cells, ***p ≤ 0.001).

The effect of Azo-NZ1 at α2Z GlyRs was drastically different from the one observed at α1Z and α1H GlyRs. The current amplitude was strongly inhibited by application of Azo-NZ1 (50 μm) in *trans*-conformation, to 9 ± 5% (*n* = 3; Fig. 3*A*,*C*), while the effect was virtually abolished on illumination with UV light. Contrary to α1 GlyRs, *trans*-Azo-NZ1 still strongly inhibited ionic currents when it was co-applied with saturating concentration of glycine. Under visible light current amplitude decreased to 24 ± 9% (*n* = 3; Fig. 3*B*,*C*).

To further clarify the properties of the Azo-NZ1, we conducted an analysis of the concentration and voltage dependencies of its inhibitory action on the α2 GlyRs. Analysis of concentration dependencies revealed that IC_50_ for Azo-NZ1 at α2Z GlyRs was 9 ± 2 μm, which was considerably lower than for α1Z GlyRs (*n* = 4; Fig. 3*D*). Voltage dependence of *trans*-Azo-NZ1 interaction with α2Z GlyRs was thus performed using 20 μm the photochrome and 50 μm glycine. At –30 mV, α2Z-mediated currents were inhibited to 49 ± 2%, while at +30 mV to 29 ± 3% (*n* = 8, *p* ≤ 0.001; Fig. 3*E*).

Voltage dependence and strong inhibitory potency even at saturating concentrations of the agonist strongly suggest that *trans-*Azo-NZ1 interacts with the ion channel pore of the receptors formed by α2 subunits.

Next, we analyzed the effect of Azo-NZ1 on heteromeric α2M/βM GlyRs. Analysis of concentration-dependencies revealed that the incorporation of β subunit lowers the sensitivity of α2 receptors to glycine. The EC_50_ for heteromeric α2M/βM GlyRs was 133 ± 20 μm (*n* = 7; Fig. 1*C*). Hence, analysis of the action of Azo-NZ1 on this heteromeric GlyR was conducted using 100 μm glycine as “non-saturating” concentration.

In the presence of 50 μm Azo-NZ1, glycine-induced currents mediated by α2M/βM GlyRs were inhibited to 48 ± 6% (*n* = 8), while currents mediated by homomeric α2M GlyRs were inhibited to 23 ± 3% (*n* = 10; Fig. 3*F*,*G*). These data suggest that incorporation of the β subunit causes a decrease in the inhibitory action of Azo-NZ1. Illumination with UV light, converting Azo-NZ1 to *cis*-form, caused recovery of the current amplitudes to nearly control values. Interestingly, in some experiments, during the first moments of the UV illumination, the current increased to values higher than predicted, showing a transient “potentiation,” which could be masked by desensitization.

### Single-channels analysis of Azo-NZ1 interaction with GlyRs

**Figure 4.**
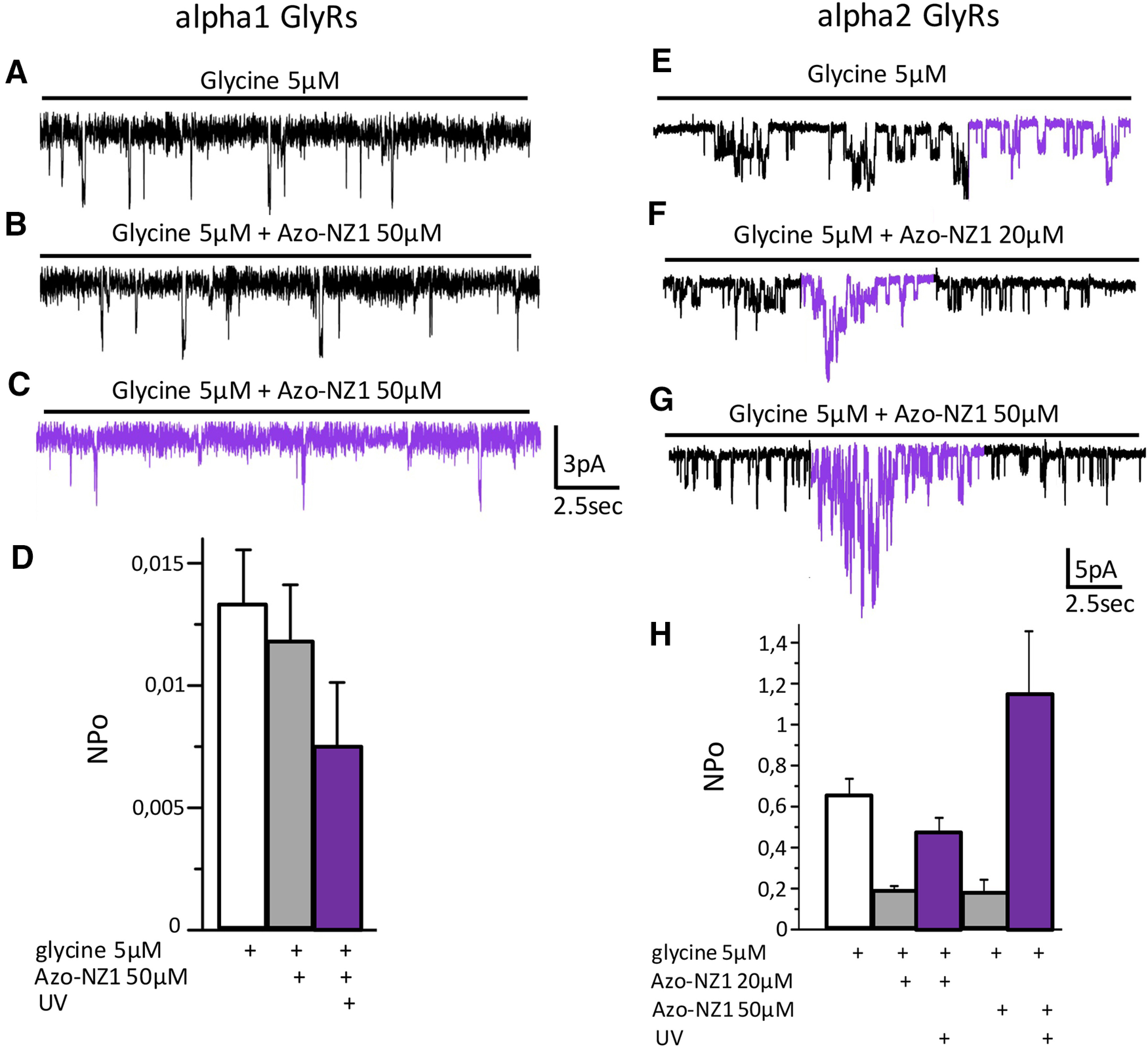
Single-channels recording of α1H and α2M GlyRs interaction with Azo-NZ1 in outside-out configuration of patch-clamp technique. ***A***, Representative recording of single-channel activity of α1Z GlyRs in control, currents were induced by application of 5 μm glycine, V_hold_ = –30 mV. ***B***, Channel activity in the solution containing a mixture of glycine 5 μm with *trans*-Azo-NZ1 50 μm at visible light and (***C***) on UV illumination (*cis*-Azo-NZ1). ***D***, NP_o_ for α1H GlyRs in control conditions (white column), when glycine 5 μm was co-applied with Azo-NZ1 50 μm under visible light (gray column), or on UV illumination (violet column). ***E***, Channel activation was induced by application of 5 μm glycine, V_hold_ = –30 mV. ***F***, A mixture of glycine 5 μm with Azo-NZ1 20 μm was applied, V_hold_ = –30 mV; the part of the trace recorded on illumination with UV light is colored in violet. ***G***, A mixture of glycine 5 μm with Azo-NZ1 50 μm was applied, V_hold_ = –30 mV; the part of the trace recorded on UV illumination is colored in violet. ***H***, NP_o_ for α2M GlyRs in control conditions (white column), when glycine 5 μm was co-applied with Azo-NZ1 20 μm or 50 μm under visible light (gray columns), or on UV illumination (violet columns).

In order to resolve in more detail the action of Azo-NZ1 on GlyR, we performed single-channel recordings of α1H and α2M GlyRs from membrane patches in outside-out configuration on application of 5 μm glycine (Fig. 4). In the control conditions, when only glycine was applied, UV illumination did not change the activity of GlyR channels (Fig. 4*E*).

In outside patches expressing α1H GlyRs, application of Azo-NZ1 (50 μm) in *trans*-state provoked a slight decrease of the open probability (NP_o_) of the channels (from 0.013 ± 0.002 to 0.011 ± 0.002), which was strengthened by UV illumination (to 0.007 ± 0.002; Fig. 4*A–D*). On outside-out patches expressing α2M GlyRs, Azo-NZ1 (20 and 50 μm) in *trans*-state induced a prominent decrease of the open probability of channels, which was accompanied by an increase in the flickering of channels openings. UV illumination caused restoration of NP_o_ and even its transient increase, particularly, in the case of application of Azo-NZ1 at 50 μm concentration. We have estimated NP_o_ of α2 GlyR channels in all conditions: in control, it was 0.64 ± 0.09; on co-application of glycine with 20 μm Azo-NZ1, it decreased to 0.19 ± 0.02; UV light restored NP_o_ almost to the control level, to 0.47 ± 0.07. Application of Azo-NZ1 in higher concentration (50 μm) decreased NP_o_ to 0.18 ± 0.06, while UV light increased it to 1.14 ± 0.3 (Fig. 4*E–H*).

These results confirm the observations obtained at whole-cell recordings that on α2 GlyRs transitions of Azo-NZ1 from *trans*- to *cis*-configuration results in transient potentiation of glycine-induced currents. Molecular basis of this phenomenon needs further analysis.

### Effect of Azo‐NZ1 on synaptic GlyRs in hypoglossal motoneurons of brainstem slices

We next asked, whether Azo-NZ1 will modulate in a light-dependent manner the function of synaptic glycinergic currents. For this analysis, hypoglossal motoneurons in brainstem slices, possessing powerful glycinergic synaptic inputs (Mukhtarov et al., 2005) were selected. As experiments with heterologous expression of different GlyR subunits demonstrated that Azo-NZ1 strongly modulates function of heteromeric α2/β GlyR channels subunits, while being weakly efficient on the α1/β GlyRs, we performed analysis on brain slices from young mice (P4–P8), expressing at this age both α1 and α2 GlyR subunits (Malosio et al., 1991; Singer et al., 1998).

**Figure 5.**
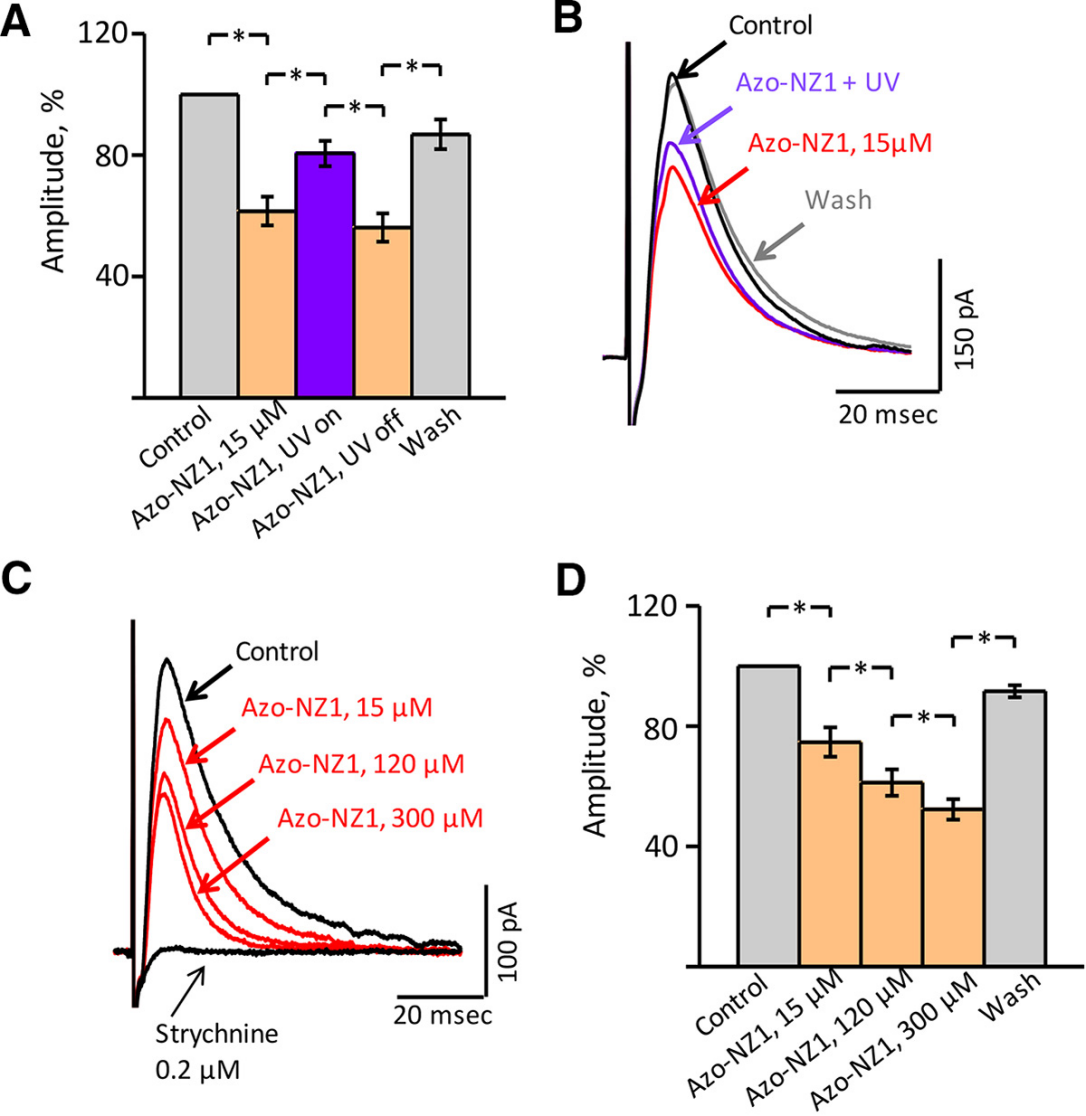
Azo-NZ1 is a photoswitchable modulator of the evoked glycinergic IPSCs (eIPSCs) in the mouse hypoglossal motoneurons. ***A***, Relative eIPSC amplitudes under the action of Azo-NZ1 (15 μm) at visible light and on UV illumination, summary of data from eight neurons (mean percentage ± SEM). *, significant difference with *p* < 0.05 (paired sample Wilcoxon signed-rank test). ***B***, Superimposed traces of eIPSCs in control condition, after addition to aCSF of 15 μm Azo‐NZ1, during illumination of Azo‐NZ1 containing aCSF by UV (365 nm) and after washing. Each trace represents the average of 18 individual eIPSCs, obtained from a P8 mouse. ***C***, Superimposed traces illustrating an example of eIPSC amplitudes decreasing under 15, 120, and 300 μm Azo‐NZ1 and complete suppression of events by 0.2 μm strychnine. Each trace represents average of 12 individual eIPSCs, obtained from a P6 mouse. ***D***, Decreasing of glycinergic eIPSC amplitude under the action of Azo-NZ1 at the concentrations of 15, 120, and 300 μm, summary of data from six neurons (mean percentage ± SEM). *, significant difference with *p* < 0.05 (paired sample Wilcoxon signed-rank test).

The effect of Azo-NZ1 on the amplitude of evoked glycinergic IPSCs (eIPSC) was analyzed in the presence of glutamate receptor and GABA_A_ receptor antagonists. In control conditions, the amplitude of eIPSCs varied from 40 to 800 pA in the different cells. The effects of Azo-NZ1 on the glycinergic eIPSCs in the mouse hypoglossal motoneurons are summarized in Figure 5. Addition of Azo-NZ1 at 15 μm concentration caused a decrease of the mean amplitude of eIPSCs to 61.5 ± 4.7% compared with control after illumination of aCSF with Azo‐NZ1 by UV, relative amplitude increased to 80.5 ± 4.2%, while under visible light it decreased to 56.1 ± 4.6% and during washing by control solution it recovered to 86.8 ± 4.9% (*n* = 8; Fig. 5*A*,*B*). Strychnine (0.2 μm) completely abolished eIPSCs, confirming the glycinergic nature of the currents (Fig. 5*C*).

Analysis of the effect of different *trans*-Azo-NZ1 concentrations on glycinergic eIPSCs showed that the amplitude of currents decreased with elevation of the doses. However, the inhibition did not exceed 50% even at high concentrations of the compound (Fig. 5*C*,*D*). Thus, the mean relative amplitudes were 61.2 ± 4.4% and 52.3 ± 3.4% in the presence of, respectively, 120 and 300 μm *trans-*Azo-NZ1 (*n* = 6).

### Mutagenesis analysis of the Azo-NZ1 interaction site at GlyRs

Previously, Azo-NZ1 was shown to be a pore-blocker of GABARs (Maleeva et al., 2019). The high level of homology between α2 GlyRs channels and GABARs at the level of TM2 helix, as well as the single-channel recordings demonstrating increased flickering of α2 channels on application of *trans*-Azo-NZ1 (Fig. 4*E–H*), suggests that Azo-NZ1 interacts with the pore of α2 GlyRs and that this interaction is strongly reduced in α1 GlyRs.

**Figure 6.**
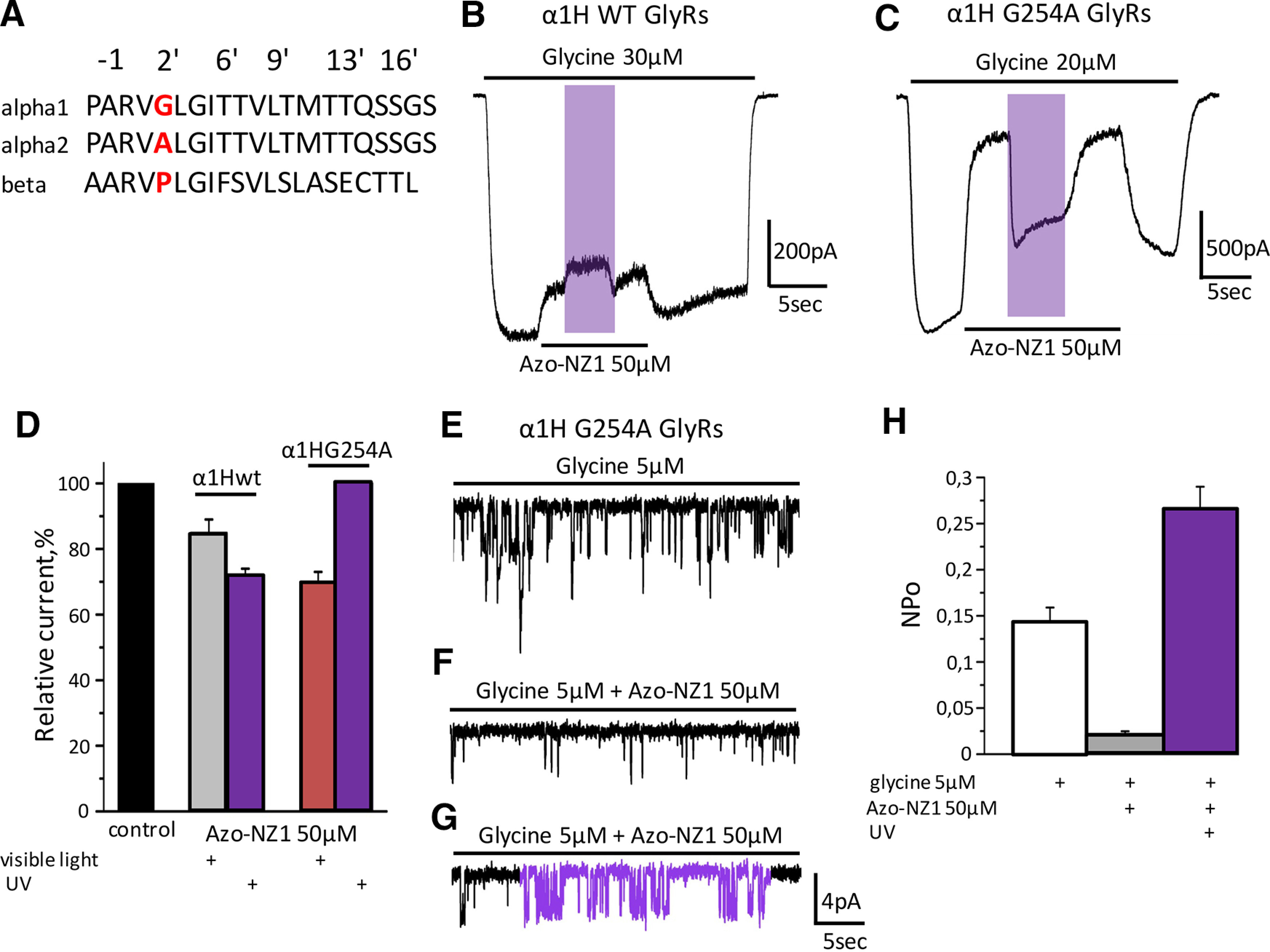
Comparison of the interaction of α1 WT GlyRs and α1 G254A GlyR with Azo-NZ1. ***A***, Amino acid sequences of TM2 domains of α1, α2, and β subunits of GlyR. ***B***, Representative trace of the α1H GlyR current induced by application of glycine 30 μm and by a mixture of glycine 30 μm with Azo-NZ1 50 μm at visible light and on UV illumination. ***C***, Representative trace of the α1H G254A GlyRs current induced by application of glycine 20 μm and by mixture of glycine with Azo-NZ1 50 μm at visible light and on UV illumination. ***D***, Relative amplitudes of α1H and α1H G254A GlyR currents in control (black column), and during application of Azo-NZ1 50 μm at visible light (gray and red columns, respectively) and at UV illumination (violet columns). ***E***, Representative single-channel recording of α1H G254A currents in control; during application of Azo-NZ1 50 μm at visible light (***F***); on illumination with UV light (***G***). ***H***, NP_o_ for α1H G254A GlyRs in control conditions (white column), when glycine 5 μm was co-applied with Azo-NZ1 50 μm under visible light (gray column) or on UV illumination (violet column).

It is well established that the amino acid sequences of ion pore-forming transmembrane domains (TMDs) of α1 and α2 subunits differ by one amino acid, which results in subunit-specific action of such inhibitory compounds as NFA and cyanotriphenylborate (CTB; Rundström et al., 1994; Maleeva et al., 2017). To clarify whether Azo-NZ1 interacts with the pore of α2 GlyRs, we generated mutant G254A α1 receptors that contained an amino acid substitution at position 2’ in the pore-forming TM2 domain. Amino acid sequences of TM2 domains of α1 and α2 subunits differ only in this position, thus G254A substitution converts the pore of α1 receptor into the one of α2. As illustrated in Figure 6*B*,*C*, the profile of α1H G254A GlyRs interaction with Azo-NZ1 was distinct from the one of α1H and Z GlyRs (Fig. 2) and closely resembled that of α2M and Z receptors (Fig. 3). The amplitude of glycine-evoked currents decreased on *trans*-Azo-NZ1 application to 70 ± 3% (*n* = 7). This effect was abolished by UV illumination (Fig. 6*B–D*), similarly to what was seen at α2M GlyRs (Fig. 3).

These results were confirmed in a series of experiments on single-channel recordings from outside-out patches expressing α1H G254A mutant GlyRs. Application of Azo-NZ1 (50 μm) decreased NP_o_ of α1H G254A channels from 0.14 ± 0.019 to 0.022 ± 0.002, while UV light induced elevation of channels activity, and NP_o_ increased 10-fold to 0.26 ± 0.025 (Fig. 6*E–H*). Thus, our single-channel experiments reproduced results obtained by whole-cell recordings, confirming a crucial role of the alanine ring at the 2’ position of GlyR channel pore in the different interaction of Azo-NZ1 with α1 and α2 GlyRs and in the different UV effect registered for these receptors.

### Modelling of Azo-NZ1 molecular interactions with GlyR

**Figure 7.**
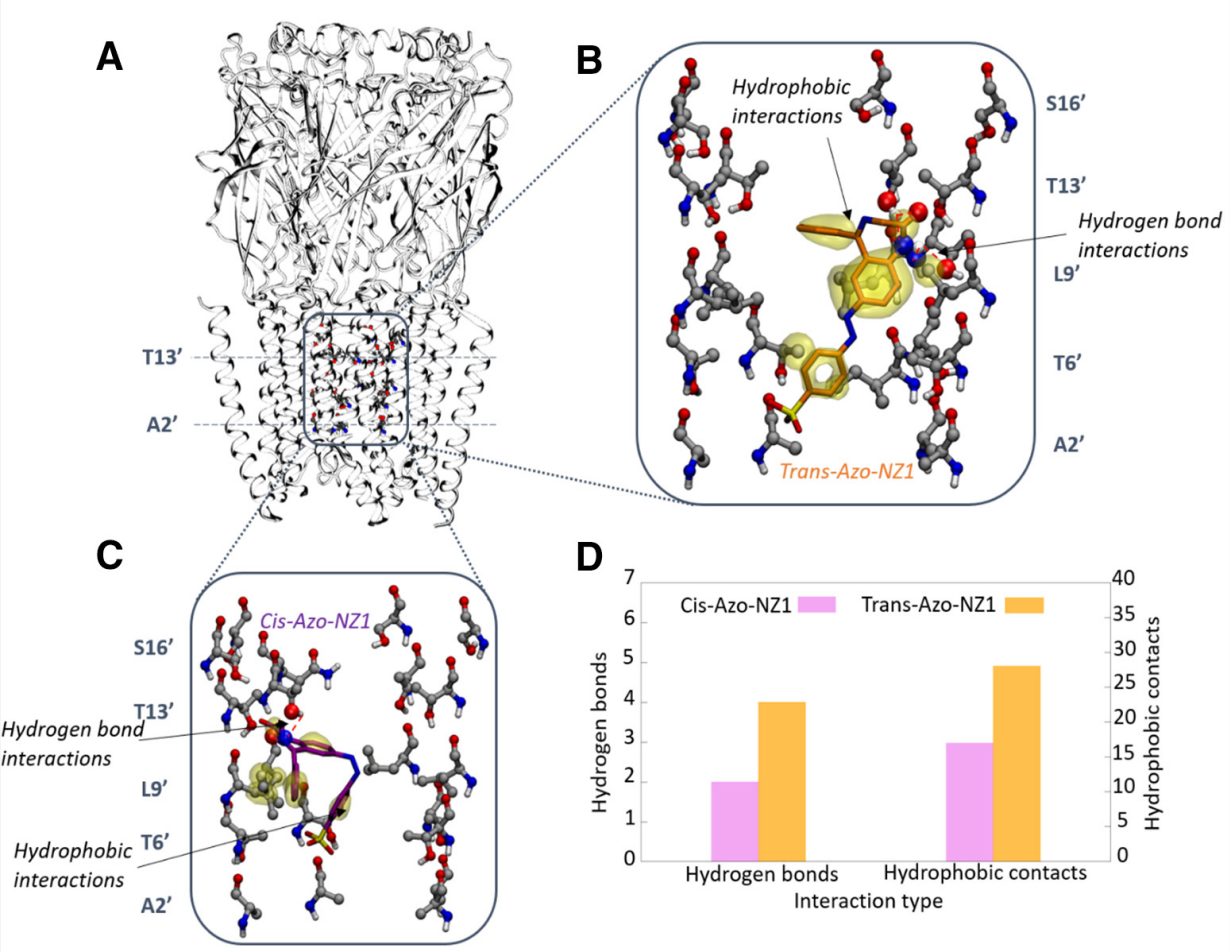
Results of the docking simulation of Azo-NZ1 in the transmembrane part domain of the α1 G254A GlyR mutant mimicking the α2 GlyR pore. Results for wild-type α1 GlyR and the heteromeric α2/β GlyR are shown in Extended Data Figures 7-1, 7-2, 7-3, 7-4, 7-5, 7-6. ***A***, Tridimensional view of the α1 G254A mutant GlyR structure (open state). The A2’ and T13’ positions are marked with dashed lines. The sulfonate group of Azo-NZ1 can bind with two orientations: up (bound at 13’) and down (bound at 2’); see Extended Data Figures 7-1, 7-3. ***B***, Binding pose of the *trans* isomer in the transmembrane domain. The sulfonate group is in the *down* configuration; see Extended Data Figure 7-3 for the corresponding poses in wild-type α1 GlyR and the heteromeric α2/β GlyR. Hydrophobic interactions between Azo-NZ1 and the receptor residues are marked as yellow transparent surfaces and hydrogen bond interactions are represented with red dashed lines. ***C***, Binding pose of the *cis* isomer in the transmembrane domain. The sulfonate group is in the down configuration; see Extended Data Figure 7-6 for the corresponding poses in wild-type α1 GlyR and the heteromeric α2/β GlyR. The representations used for hydrophobic and hydrogen bond interactions are the same as for the *trans* isomer. ***D***, Relative number of intermolecular interactions formed by the *cis* and *trans* isomers of Azo-NZ1 with the pore-lining residues in α1 G254A mutant GlyR.

To understand the mechanism of action of Azo-NZ1 at atomic level, we performed molecular docking calculations for three different GlyRs: homomeric α1Z wild-type, α1Z G254A mutant (equivalent to α2Z in the pore) and heteromeric α2M/βM (see Materials and Methods). All receptors were modelled in the open channel state. In view of the importance of the 2’ pore-lining amino acids indicated by the experimental data (Fig. 6), the docking simulation was conducted in the transmembrane region of the channel pore (Fig. 7*A*).

10.1523/ENEURO.0294-20.2020.f7-1 Extended Data Figure 7-1 Density map of the sulfonate group of *trans*-Azo-NZ1 bound in the transmembrane part of the pore of α1, α1G254A, and α2/β GlyRs. Regions of continuous density correspond to higher sulfonate occupancy and indicate tighter sulfonate binding. Each contour line corresponds to one particle/nm^3^, where “particle” refers to the sulfonate group in a given binding pose and is used to follow Azo-NZ1 binding. Longitudinal view of the pore: the front subunit is not displayed to reveal the interior of the pore. The S/T16’ and G/A/P2’ regions are marked with dashed lines. A higher percentage of binding poses is observed in these two regions; the percentage of poses (between parentheses) where the sulfonate is placed in the 16’ region (up configuration) is 40%, 47%, and 34%, whereas 21%, 21%, and 26% of binding poses placed the sulfonate in the 2’ region (down configuration) for α1, α1G254A, and α2/β GlyRs, respectively. Download Figure 7-1, TIF file.

10.1523/ENEURO.0294-20.2020.f7-2 Extended Data Figure 7-2 Interactions of *trans*-Azo-NZ1 in the “up” binding pose for (***A***) α1, (***B***) α1G254A, and (***C***) α2/β GlyRs. A longitudinal view of the pore is shown, with the pore lining residues represented as ball-and-sticks and their carbon atoms colored in gray. *Trans-*Azo-NZ1 is represented as sticks with carbon atoms in orange. Nitrogen and oxygen atoms are colored in blue and red, respectively. Hydrogen bonds are marked with red dashed lines and hydrophobic interactions are represented as yellow surfaces. Download Figure 7-2, TIF file.

10.1523/ENEURO.0294-20.2020.f7-3 Extended Data Figure 7-3 Interactions of *trans*-Azo-NZ1 in the “down” binding pose for (***A***) α1, (***B***) α1G254A, and (***C***) α2/β GlyRs. Representations and color code are the same as in Extended Data Figure 7-2. Download Figure 7-3, TIF file.

10.1523/ENEURO.0294-20.2020.f7-4 Extended Data Figure 7-4 Density map of the sulfonate group of *cis*-Azo-NZ1 bound in the transmembrane part of the pore for (***A***) α1, (***B***) α1G254A, and (***C***) α2/β GlyR’s. A longitudinal view of the pore is shown; the front subunit is not displayed to reveal the interior of the pore. The T/S13’ and G/A/P2’ regions are marked with dashed lines. A higher percentage of binding poses is observed in the 13’ region for the first two receptors, while for the heteromeric α2/β GlyR binding poses are more dispersed all over the 16’–2’ region. The percentage of binding poses (between parentheses) where the sulfonate is placed in the 13’ region is 60%, 60%, and 65% for α1, α1G254A, and α2/β GlyR’s, respectively. Only the the heteromeric α2/β GlyR shows a significant percentage of poses with the sulfonate bound at 2’ (down configuration), i.e., 34%. Download Figure 7-4, TIF file.

10.1523/ENEURO.0294-20.2020.f7-5 Extended Data Figure 7-5 Interactions of *cis*-Azo-NZ1 in the “up” binding pose for (***A***) α1, (***B***) α1G254A, and (***C***) α2/β GlyRs. A longitudinal view of the pore is shown, with the pore lining residues represented as ball-and-sticks and their carbon atoms are colored in grey. *Cis*-Azo-NZ1 is represented as sticks with carbon atoms in purple. Nitrogen and oxygen atoms are colored in blue and red, respectively. Hydrogen bonds are marked with red dashed lines and hydrophobic interactions are represented as yellow surfaces. Download Figure 7-5, TIF file.

10.1523/ENEURO.0294-20.2020.f7-6 Extended Data Figure 7-6 Interactions of *cis*-Azo-NZ1 in the “down” binding pose for (***A***) α1, (***B***) α1G254A, and (***C***) α2/β GlyRs. Representations and color code are the same as in Extended Data Figure 7-5. Note that there is no significant sulfonate density at position 2’ for wild-type α1 GlyR (Extended Data Figure 7-4*A*), and thus, we expect that the probability of having *cis*-Azo-NZ1 bound in the down configuration for this receptor is very low. Download Figure 7-6, TIF file.

10.1523/ENEURO.0294-20.2020.f7-7 Extended Data Figure 7-7 Density map of the sulfonate group of Azo-NZ1 resulting from the blind docking to (***A***, ***B***) α1 and (***C***, ***D***) α2 GlyRs. Regions of continuous density correspond to higher sulfonate occupancy and indicate tighter sulfonate binding. Each contour line corresponds to 0.0006 particle/nm^3^, where “particle” refers to the sulfonate group in a given binding pose and is used to follow the putative interaction regions of Azo-NZ1. Longitudinal view of the receptor: the front subunit is not displayed for the sake of clarity. The pore region (between positions S/T16’ and G/A2’), as well as the interface region between the ECD-TMD, are marked with dashed lines. The residue at position 2’ is displayed as black spheres. *Trans*-Azo-NZ1 binds preferentially inside the 16’–2’ region for both GlyRs (37% and 46% for α1 and α2 GlyRs, respectively). Instead, *cis*-Azo-NZ1 shows two possible interaction sites, either inside the pore or at the ECD-TMD interface. The relative percentage of poses in the two regions varies significantly between the two GlyR types. For α1 GlyR, *cis*-Azo-NZ1 has a higher probability to interact with the ECD-TMD interface (35%) than with the pore (28%). In contrast, for α2 GlyR, the sulfonate density in the interface region is more dispersed, and the associated probability is lower (28%) than the one corresponding to binding in the pore (46%). Download Figure 7-7, TIF file.

The docking results for *trans*-Azo-NZ1 show a similar binding pattern for all three GlyRs (Extended Data Fig. 7-1). *Trans*-Azo-NZ1 binds preferably between the 2’−16’ pore region, with two possible orientations of the sulfonate group (Extended Data Figs. 7-2, 7-3): either with the sulfonate group between the 13’−16’ rings (hereafter, up configuration) or with sulfonate in the 2’ region (i.e., down configuration).

In contrast, the sulfonate density of *cis*-Azo-NZ1 was found to be more dispersed (Extended Data Fig. 7-4), indicating weaker binding. Moreover, it is mainly concentrated in the 6’−13’ region (Extended Data Fig. 7-5), where the diameter of the pore is wider and thus more difficult to block completely. Analysis of the binding pose of the *cis* isomer (Fig. 7*C*; Extended Data Fig. 7-6) shows that, because of its staple-like shape, the ligand is slightly shifted upwards with respect to the *trans* isomer and thus it cannot block chloride conduction as effectively as the *trans* isomer (either sterically or electrostatically). Moreover, the number of interactions with the receptor is decreased for *cis*-Azo-NZ1 compared with the *trans* isomer (Fig. 7*D*), indicating that the ligand will have a higher probability of dissociation on UV irradiation. Therefore, our simulations predict that on isomerization, even if *cis*-Azo-NZ1 remains bound, it would be less capable to block the pore of the GlyRs compared with the *trans* isomer.

Out of the two aforementioned orientations of *trans*-Azo-NZ1 in the pore, the up pose is unlikely to block conduction. On one hand, the negatively charged sulfonate group is located in the wider 16’ region and thus would not be able to electrostatically hinder the chloride ion flow. On the other, the ligand also has a lower number of interactions with the lining pore residues (either hydrogen bond interactions or hydrophobic ones), indicating weaker binding. Hence, we will focus on the *trans*-Azo-NZ1 docking poses (Fig. 7*B*) where the sulfonate group is placed in the narrower 2’ pore region (down), as it seems to be the most likely blocking site for *trans*-Azo-NZ1. Such a ligand orientation is stabilized by multiple hydrogen bond interactions of the amide group of the nitrazepam core of Azo-NZ1 with the hydroxyl group of one of the five T13’ residues, as well as with the backbone of T10’ residues. Further stabilization is provided by the hydrophobic contacts involving the apolar region of the nitrazepam core, the side chain of L9’ and the methyl group of T6’ (Fig. 7*B*). The length of the ligand (14.1 Å) is optimal to place the polar amide group close to the polar T13’ side chain and, most importantly, the negatively charged sulfonate group is placed in the more constricted region of the pore, the 2’ ring, since T13’ and A2’ are ∼16 Å apart. Thus, the hydrogen bond seen with the 2’ residue in GABA_A_ and GABA rho2 receptors (Maleeva et al., 2019) is important for orienting the ligand with the sulfonate toward the extracellular part of the receptor, but it is not essential for inhibition, as it can be seen with these newest results on GlyRs. Indeed, sulfonate binding at the 2’ position is possible as long as the 2’ ring has the right diameter to accommodate the sulfonate group. In this regard, the volume of A2’ in α1 G254A GlyR is similar to that of S2’ in GABA rho2 and the γ subunit of GABA_A_ receptors. Moreover, the interactions with other pore-lining residues (6’, 9’, and 13’) appear to have a stronger contribution to the binding of *trans*-Azo-NZ1 in GlyRs than in GABARs.

**Figure 8.**
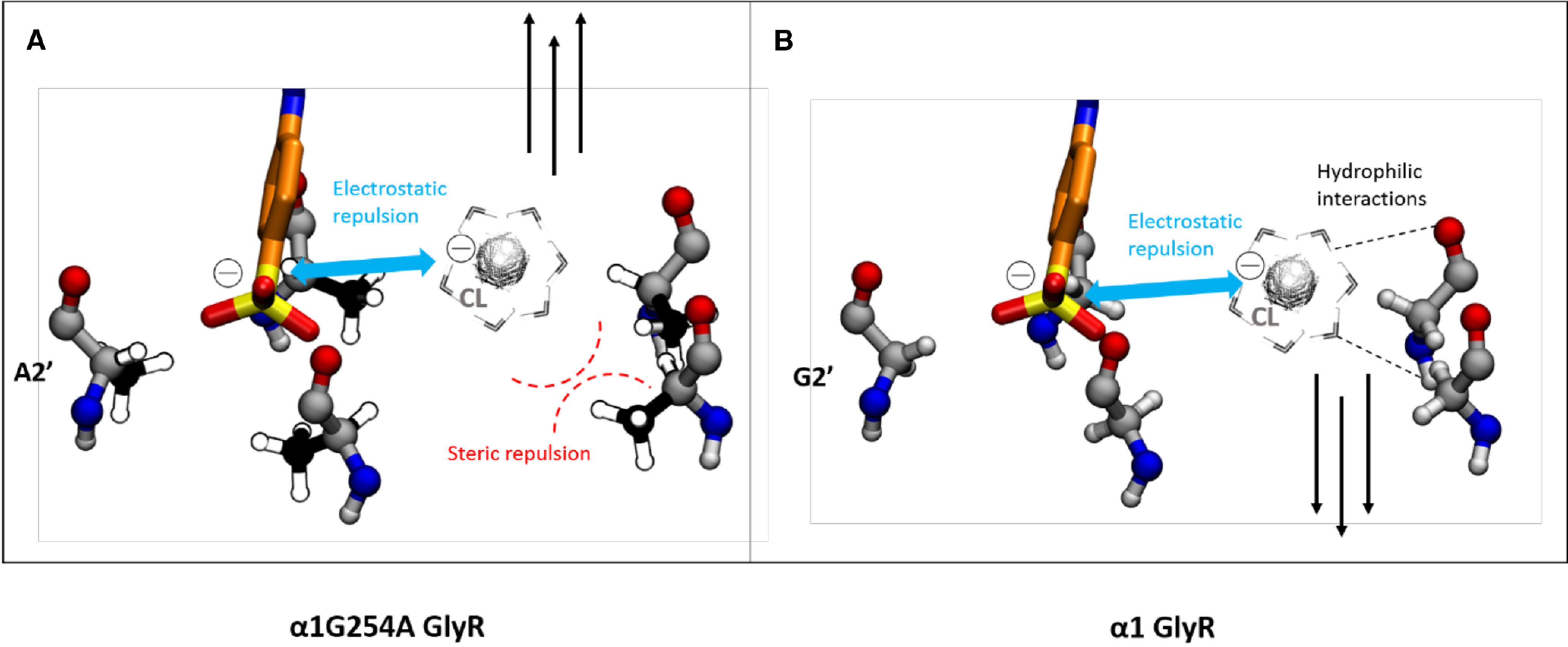
Proposed scheme of the passage of a hydrated chloride ion through the 2’ pore region in α1 G254A and α1 GlyRs. The hydrated chloride ion is represented in greyscale. Electrostatic repulsion between the negatively charged sulfonate group and the chloride ion is represented with a blue arrow, the steric hindrance between the chloride ion and the methyl side chain of A2’ is shown as curved red dashed lines, and the hydrophilic interactions between the water molecules in the chloride ion solvation shell and the G2’ backbone are represented as black dashed lines. ***A***, In the α1G254A GlyR mutant, the electrostatic and steric barrier created by the combined effect of the sulfonate group of *trans*-Azo-NZ1 and A2’, respectively, does not let the chloride ion go through this narrow part of the pore. ***B***, In wild-type α1 GlyR, the electrostatic repulsion with the sulfonate group is not enough to prevent chloride conduction, as the residue at the 2’ position is now glycine, so that the pore has more space and the chloride ion can also form hydrophilic interactions with the G2’ backbone.

The only difference between α1 and α2 Gly receptors in the pore channel is the change from glycine to alanine. This minor change produces different inhibitory activities for both α GlyRs, mainly decreasing the inhibitory effect of *trans*-Azo-NZ1 for α1 GlyR. The Ala2’ residue in α2 GlyR is placing five methyl groups, one from each of the five M2 helixes, in the narrowest part of the pore, compared with five hydrogen atoms for the Gly2’ ring in α1 GlyR. This decreases the diameter of the pore at the 2’ position by at least 1 Å in α2 GlyR, and thus makes it easier for the sulfonate group to clog the pore. Interestingly, the negatively charged sulfonate group of *trans*-Azo-NZ1 (down pose) occupies the same position as the chloride ion trapped in x-ray structures of GABA_A_R (Laverty et al., 2017) and GlyR (Huang et al., 2017). Thus, Ala2’ residues add steric hindrance (Fig. 8*A*), which would be another obstacle for the hydrated chloride ion, besides the electrostatic repulsion with the sulfonate group of *trans*-Azo-NZ1. In contrast, Gly2’ loses this steric repulsion (Fig. 8*B*) and potentially adds a favorable ion-dipole interaction with chloride, since its backbone is more exposed into the pore (Sunesen et al., 2006).

The results for the heteromeric α2/β GlyR are similar to the G254A α1 mutant GlyR, for both *trans*- and *cis*-Azo-NZ1 conformers (Extended Data Figs. 7-3, 7-6). In particular, the down binding pose of *trans*-Azo-NZ1 is almost identical for α1 G254A and α2/β GlyRs (Extended Data Fig. 7-3*B*,*C*), in agreement with the experimental observation of both receptors being inhibited by the *trans* isomer. In other words, although the replacement of two Ala residues in the 2’ ring (α1 G254A and α2) by Pro (β) reduces the diameter of the pore at this position by ∼2 Å, it still allows sulfonate binding.

The docking results obtained rationalize the stronger blocking effect of *trans*-Azo-NZ1 on α2 GlyRs compared with α1 GlyR and the relief of the pore blocking-mediated inhibition of α2 GlyRs on *cis* isomerization. However, α1 GlyRs show an unexpected behavior, in that they are weakly inhibited by *trans*-Azo-NZ1 but inhibition increases slightly on UV irradiation (Fig. 2). Considering our docking results for *cis*-Azo-NZ1 inside the pore (Extended Data Figs. 7-4*A*, 7-5*A*) and the lack of inhibition of α1 GlyR at saturating glycine concentrations (Fig. 2*B*), we explored other possible interaction sites of *cis*-Azo-NZ1 outside the M2 pore. Our results (Extended Data Fig. 7-7) suggest that *trans*-Azo-NZ1 binds preferentially inside the pore (Extended Data Fig. 7-7*C*,*D*), whereas *cis*-Azo-NZ1 might bind in both the M2 pore-lining region and an alternative site at the interface between the extracellular domain (ECD) and TMD (Extended Data Fig. 7-7*A*,*B*), analogously to another azo-nitrazepam-based GlyR inhibitor, Glyght (Gomila et al., 2020). Moreover, *cis-*Azo-NZ1 has a slightly higher probability to interact with ECD-TMD region (35%) than the pore (28%) in α1 GlyR, whereas in α2 GlyR it mostly binds in the pore (46%). Altogether, we surmise that *cis*-Azo-NZ1 might be able to inhibit α1 GlyR by binding to the ECD-TMD interface.

## Discussion

This study presents the experimental and modeling analysis of the action of Azo-NZ1 on α1 and α2 subunits of GlyR heterologously expressed in cultured cells and also on glycinergic synaptic currents in whole-cell recordings from hypoglossal motoneurons of brainstem slices. Our observations demonstrate that Azo-NZ1 is an efficient and subunit-specific photoswitchable inhibitor of GlyRs with the following main differences between its action on α1 and α2 GlyR subunits: (1) in *trans*-configuration, Azo-NZ1 caused strong inhibition of glycine-induced currents on cells expressing homomeric α2 GlyRs, while it was not able to modulate α1 GlyRs responses at saturating concentrations of glycine; (2) in *cis*-configuration, induced by UV illumination, Azo-NZ1 completely lost the ability to inhibit α2 GlyRs.

The aforementioned differences in the interaction of Azo-NZ1 with α1 and α2 GlyRs, as well as the fact that the sensitivity of α2 GlyRs to Azo-NZ1 (IC_50_ = 9 μm) is much higher than the one of GlyRα1 and GABA_A_Rs (IC_50_ = 67 μm; Maleeva et al., 2019), suggest that this photochromic compound might be used as selective light-dependent modulator of α2-containing GlyRs.

Extrasynaptic homomeric GlyRα2 or GlyRα3 were detected in the striatum, hippocampus, and prefrontal cortex (McCracken et al., 2017), and in neurons of the trigeminal mesencephalic nucleus (Bae et al., 2018). Tonically active GlyRs also regulate the firing of medium spiny neurons of the dorsal striatum and may thus affect the function of basal ganglia (Molchanova et al., 2018). As the TM2 domains, forming the ion selective pore is identical for GlyRα2 and GlyRα3, these extrasynaptic receptors tonically regulating firing properties of neuronal circuits represent the important target for Azo-NZ1 action.

The properties of Azo-NZ1 suggest that this compound may represent a promising tool for the pharmacological discrimination of the extrasynaptic GlyRs and for the studying of the processes mediated by them, as well as for light-controlled regulation of neuronal plasticity and excitability in the different brain regions.

The features of Azo-NZ1 action indicate that Azo-NZ1 interacts primarily with the ion pore domain of α2 GlyRs. This assumption is supported by our experiments demonstrating that substitution of single amino acid (G254A) situated in 2’ position of the pore-forming TM2 domain of α1 receptors was sufficient to switch the mode of Azo-NZ1 action, which became similar to those observed for α2 GlyRs (Fig. 6*B*,*C*). An additional argument for the pore action of this photoswitch is provided by the comparison of Azo-NZ1 effects on homomeric and heteromeric GlyRs formed by α2 or α2/β subunits. Incorporation of β subunits, which possess proline at the 2’ position, caused a decrease in the blocking ability of Azo-NZ1 without changing the light-controlled switching (Fig. 3*F*,*G*). This is consistent with our recent observations on homomeric rho1 GABA_C_ receptors, which demonstrated that proline in 2’ position of TM2 results in the complete absence of Azo-NZ1 inhibitory effect (Maleeva et al., 2019).

The modeling of Azo-NZ1 molecular interactions with GlyR suggests that the pore blocking effect of *trans*-Azo-NZ1 is because of two essential features of the photochromic molecule. First, the negative charge of the sulfonate group, which mimics that of the chloride ion. Second, its longitude (14.1 Å) which allows Azo-NZ1 to form hydrogen bonds between the amide group of the nitrazepam core and T13’ and place the sulfonate in the 2’ region, thus spanning the whole pore (Fig. 7*B*). In the 2’ ring, the sulfonate group is able to (sterically and electrostatically) block chloride conduction depending on the 2’ residue, glycine (in α1 subunits) or alanine (in α2 subunits). With glycine, the hydrated chloride is still able to go through the 2’ ring thanks to the exposed hydrophilic backbone of glycine and the wider space in that constriction (Fig. 8*B*). In contrast, alanine has a methyl group that increases steric hindrance (together with the steric and electrostatic barrier of the sulfonate group, see Fig. 8*A*), resulting in the strong inhibition of this type of GlyR.

Differences in the gating of α1 and α2 GlyRs may play as well an important role in the interaction of Azo-NZ1 with α1 and α2 GlyRs, along with differences in the pore geometry. In our single-channel recordings, NP_o_ for α2 GlyRs in control is drastically higher than NP_o_ for α1 receptors. This is in accordance with previous works demonstrating that the mean open time of α2 channels is almost 100-fold longer than the one of α1 (Takahashi et al., 1992). Accordingly, Azo-NZ1 interaction with the channel pore of α1 GlyRs might be additionally prevented by a short channel openings characteristic of this receptor.

Several previously described inhibitors of GlyRs were shown to act through the pore of the channel. CTB blocks α1 GlyRs with high efficiency, while being two orders magnitude less efficient at α2 GlyRs (Rundström et al., 1994). Exchange of the TM2 domain of the α1 subunit by the one of α2 imparted resistance to CTB to the receptor, indicating that the negatively charged CTB acts as pore-blocker. Also, the uncharged GlyR inhibitor PTX was shown to interact with the pore of GlyRs (Zhorov and Bregestovski, 2000; Yang et al., 2007; Gielen et al., 2015; Gielen and Corringer, 2018). Its blocking ability at homomeric α1 and α2 GlyRs was similar, however, incorporation of β subunit caused a drastic decrease in PTX blocking activity on heteromeric GlyRs (Pribilla et al., 1992). It was suggested that both 6’ and 2’ amino acids of the pore are involved in binding of PTX (Yang et al., 2007).

Another inhibitor of GlyRs, NFA, was shown to block the Cl-selective ion channel (Maleeva et al., 2017). NFA has ∼10-fold higher affinity to α2 than to α1 GlyRs and G254A mutation in α1 receptors increases its sensitivity to NFA, indicating the Ala2’ residue in TM2 pore domain as the main site of its action. However, the β subunit does not have a strong impact on the interaction of NFA with GlyRs (Maleeva et al., 2017). Therefore, the 2’ amino acids ring of the TM2 pore is the key point of action of different channel blockers. Azo-NZ1 has a distinct profile of interaction with GlyRs. Similarly to NFA, it inhibits much stronger α2 receptors than α1 GlyRs, while, similarly to PTX, incorporation of β subunit decreased sensitivity of α2 receptors to Azo-NZ1. These similarities support common interaction points inside of the channel pore that Azo-NZ1 shares with PTX and NFA.

Analysis of single-channels recordings of α2 GlyRs activity on application of Azo-NZ1 disclosed another very interesting aspect of their interaction. UV illumination at high (50 μm) concentration of Azo-NZ1 produced a strong transient increase of the open probability of α2 channels. Apparently Azo-NZ1 while being switched from *trans-* to *cis*-states is able to induce elevation of the open probability of α2 channels. This effect was completely absent at application of Azo-NZ1 to α1 GlyRs, confirming the mismatch of their interaction sites. The molecular mechanism of this transient potentiation of α2 GlyRs needs to be clarified.

Our electrophysiological analysis in the mouse hypoglossal motoneuron, proved Azo-NZ1 to be a photoswitchable modulator of the glycinergic eIPSCs and further support the suggestion that the photochrome is a selective modulator of synaptic transmission mediated by α2 GlyRs. We show that at the high concentrations causing complete block of α2-containing GlyRs (Fig. 3*D*), *trans*-Azo-NZ1 inhibited the amplitude of glycinergic eIPSCs only by ∼50% (Fig. 5*C*,*D*). This is in line with earlier observations demonstrating that during the early postnatal development, the subunit composition of GlyRs in the hypoglossal motoneurons continuously changes, resulting in the decreasing of “fetal” α2 subunits and increasing of “adult” α1 subunits expression (Malosio et al., 1991; Singer et al., 1998). At the intermediate age (P5-P8) of the mice we used in the present study, the motoneurons express nearly equal proportion of α1 and α2 GlyR subunits (Singer et al., 1998). It is also well documented that the peak concentration of glycine acting on postsynaptic receptors of motoneurons is ∼3 mm (Beato, 2008). At these highly saturating agonist concentrations, α1 GlyRs are not inhibited by *trans*-Azo-NZ1 (Fig. 2*B*) and the photochrome should operate as specific blocker of α2 GlyRs and thus fetal glycinergic synapses. Another reason for the moderate blocking of eIPSCs is that they are mainly mediated by synaptic α2/β receptors, which, as we have demonstrated in heterologous expression system, are less sensitive to Azo-NZ1 inhibitory effect. Future studies should be aimed at investigating the effect of Azo-NZ1 on the tonic currents that are mediated by homomeric receptors, as Azo-NZ1 demonstrate highest affinity for α2 homomeric receptors and thus can reveal itself to be an excellent tool for unravelling their physiological role.

In conclusion, we present here the detailed analysis of the novel photochromic compound Azo-NZ1 action on different GlyRs expressed heterologously and on synaptic glycinergic currents in brain slices. Experimental observations, supported by modeling analysis, demonstrate that Azo-NZ1 is a potent TM2 pore blocker of α2 GlyRs. This subunit-specific light-controlled modulator of GlyRs may be a useful tool for controlling neuronal activity in spinal cord, brainstem, cerebellum and other parts of nervous system with high expression of glycinergic synapses.

## Synthesis

Reviewing Editor: Alexey Semyanov, Shemyakin-Ovchinnikov Institute of Bioorganic Chemistry Decisions are customarily a result of the Reviewing Editor and the peer reviewers coming together and discussing their recommendations until a consensus is reached. When revisions are invited, a fact-based synthesis statement explaining their decision and outlining what is needed to prepare a revision will be listed below. The following reviewer(s) agreed to reveal their identity: Timothy Lynagh.

Both reviewers have found your results interesting. However, they have also raised some concerns about the data presentations and discussion. For example, concentration-response curves may be useful to characterize 2' mutation. Non-synaptic receptors GlyR as a primary target for Azo-NZ1 have to be considered. Data analysis should be explained more clearly. Some discussion to what extent the modelling concords with the electrophysiology data should be included.
