# Supplementary figures and images for: Subunit-Specific Photocontrol of Glycine Receptors by Azobenzene-Nitrazepam Photoswitcher
Source: eNeuro. 2021 Jan 15;8(1):ENEURO.0294-20.2020. doi: 10.1523/ENEURO.0294-20.2020 (PMC7877471; doi:10.1523/ENEURO.0294-20.2020)

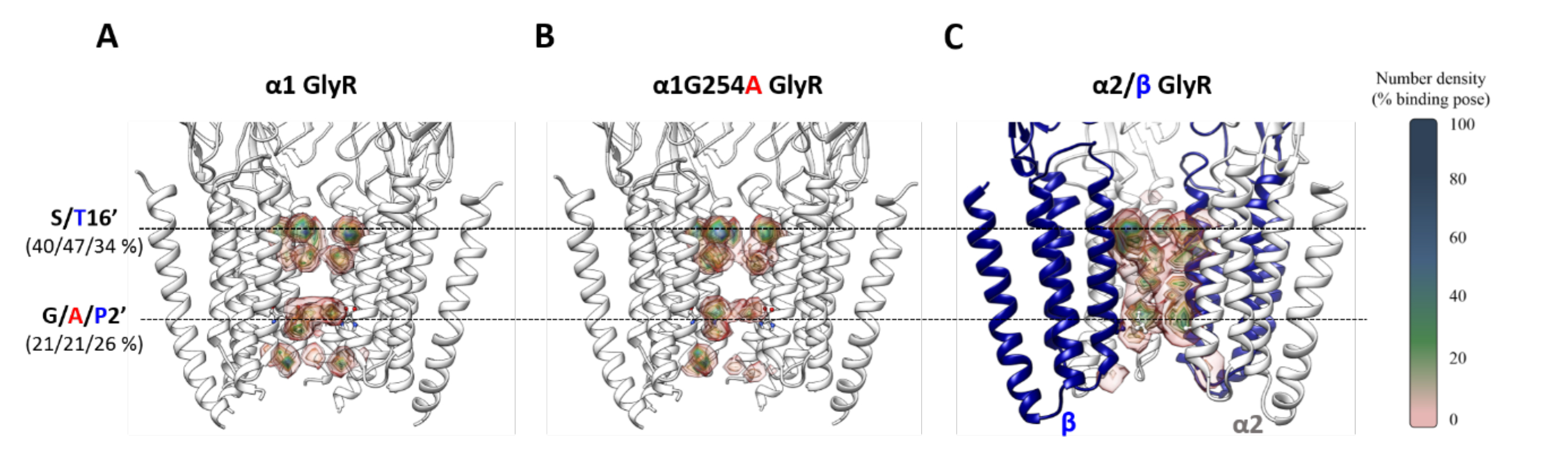

Supplement: Extended Data Figure 7-1 — Density map of the sulfonate group of trans-Azo-NZ1 bound in the transmembrane part of the pore of α1, α1G254A, and α2/β GlyRs. Regions of continuous density correspond to higher sulfonate occupancy and indicate tighter sulfonate binding. Each contour line corresponds to one particle/nm3, where “particle” refers to the sulfonate group in a given binding pose and is used to follow Azo-NZ1 binding. Longitudinal view of the pore: the front subunit is not displayed to reveal the interior of the pore. The S/T16’ and G/A/P2’ regions are marked with dashed lines. A higher percentage of binding poses is observed in these two regions; the percentage of poses (between parentheses) where the sulfonate is placed in the 16’ region (up configuration) is 40%, 47%, and 34%, whereas 21%, 21%, and 26% of binding poses placed the sulfonate in the 2’ region (down configuration) for α1, α1G254A, and α2/β GlyRs, respectively. Download Figure 7-1, TIF file. [file enu-eN-NWR-0294-20-s03.tif]

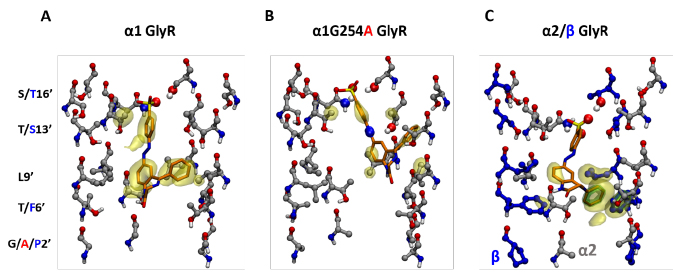

Supplement: Extended Data Figure 7-2 — Interactions of trans-Azo-NZ1 in the “up” binding pose for (A) α1, (B) α1G254A, and (C) α2/β GlyRs. A longitudinal view of the pore is shown, with the pore lining residues represented as ball-and-sticks and their carbon atoms colored in gray. Trans-Azo-NZ1 is represented as sticks with carbon atoms in orange. Nitrogen and oxygen atoms are colored in blue and red, respectively. Hydrogen bonds are marked with red dashed lines and hydrophobic interactions are represented as yellow surfaces. Download Figure 7-2, TIF file. [file enu-eN-NWR-0294-20-s04.tif]

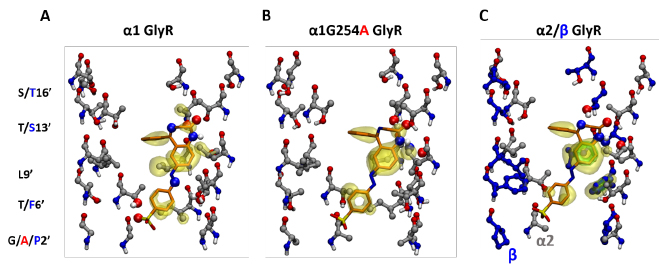

Supplement: Extended Data Figure 7-3 — Interactions of trans-Azo-NZ1 in the “down” binding pose for (A) α1, (B) α1G254A, and (C) α2/β GlyRs. Representations and color code are the same as in Extended Data Figure 7-2. Download Figure 7-3, TIF file. [file enu-eN-NWR-0294-20-s05.tif]

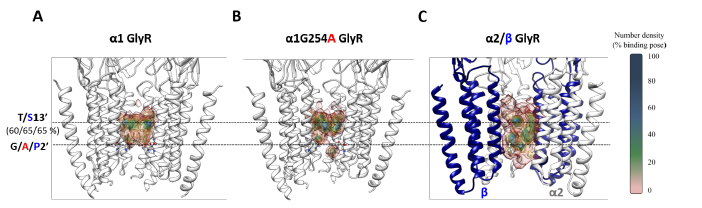

Supplement: Extended Data Figure 7-4 — Density map of the sulfonate group of cis-Azo-NZ1 bound in the transmembrane part of the pore for (A) α1, (B) α1G254A, and (C) α2/β GlyR’s. A longitudinal view of the pore is shown; the front subunit is not displayed to reveal the interior of the pore. The T/S13’ and G/A/P2’ regions are marked with dashed lines. A higher percentage of binding poses is observed in the 13’ region for the first two receptors, while for the heteromeric α2/β GlyR binding poses are more dispersed all over the 16’–2’ region. The percentage of binding poses (between parentheses) where the sulfonate is placed in the 13’ region is 60%, 60%, and 65% for α1, α1G254A, and α2/β GlyR’s, respectively. Only the the heteromeric α2/β GlyR shows a significant percentage of poses with the sulfonate bound at 2’ (down configuration), i.e., 34%. Download Figure 7-4, TIF file. [file enu-eN-NWR-0294-20-s06.tif]

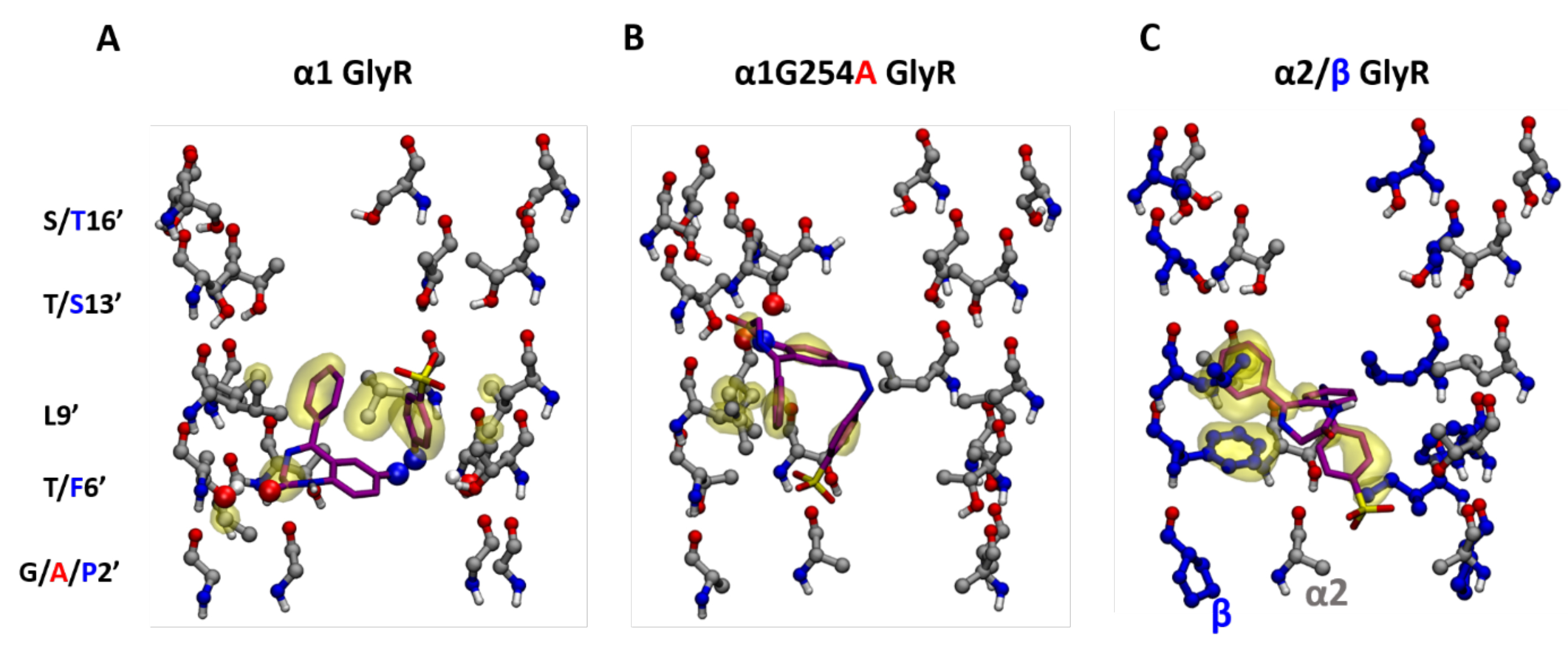

Supplement: Extended Data Figure 7-5 — Interactions of cis-Azo-NZ1 in the “up” binding pose for (A) α1, (B) α1G254A, and (C) α2/β GlyRs. A longitudinal view of the pore is shown, with the pore lining residues represented as ball-and-sticks and their carbon atoms are colored in grey. Cis-Azo-NZ1 is represented as sticks with carbon atoms in purple. Nitrogen and oxygen atoms are colored in blue and red, respectively. Hydrogen bonds are marked with red dashed lines and hydrophobic interactions are represented as yellow surfaces. Download Figure 7-5, TIF file. [file enu-eN-NWR-0294-20-s07.tif]

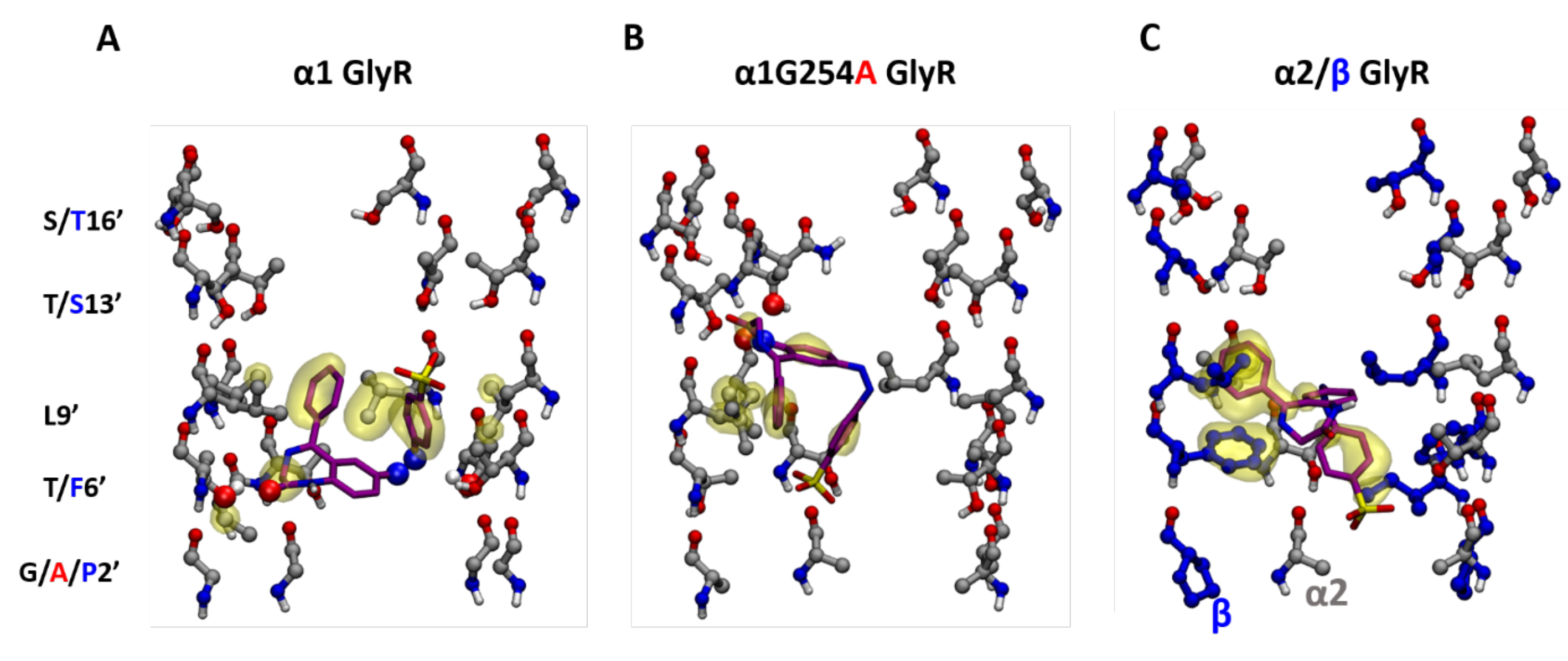

Supplement: Extended Data Figure 7-6 — Interactions of cis-Azo-NZ1 in the “down” binding pose for (A) α1, (B) α1G254A, and (C) α2/β GlyRs. Representations and color code are the same as in Extended Data Figure 7-5. Note that there is no significant sulfonate density at position 2’ for wild-type α1 GlyR (Extended Data Figure 7-4A), and thus, we expect that the probability of having cis-Azo-NZ1 bound in the down configuration for this receptor is very low. Download Figure 7-6, TIF file. [file enu-eN-NWR-0294-20-s08.tif]

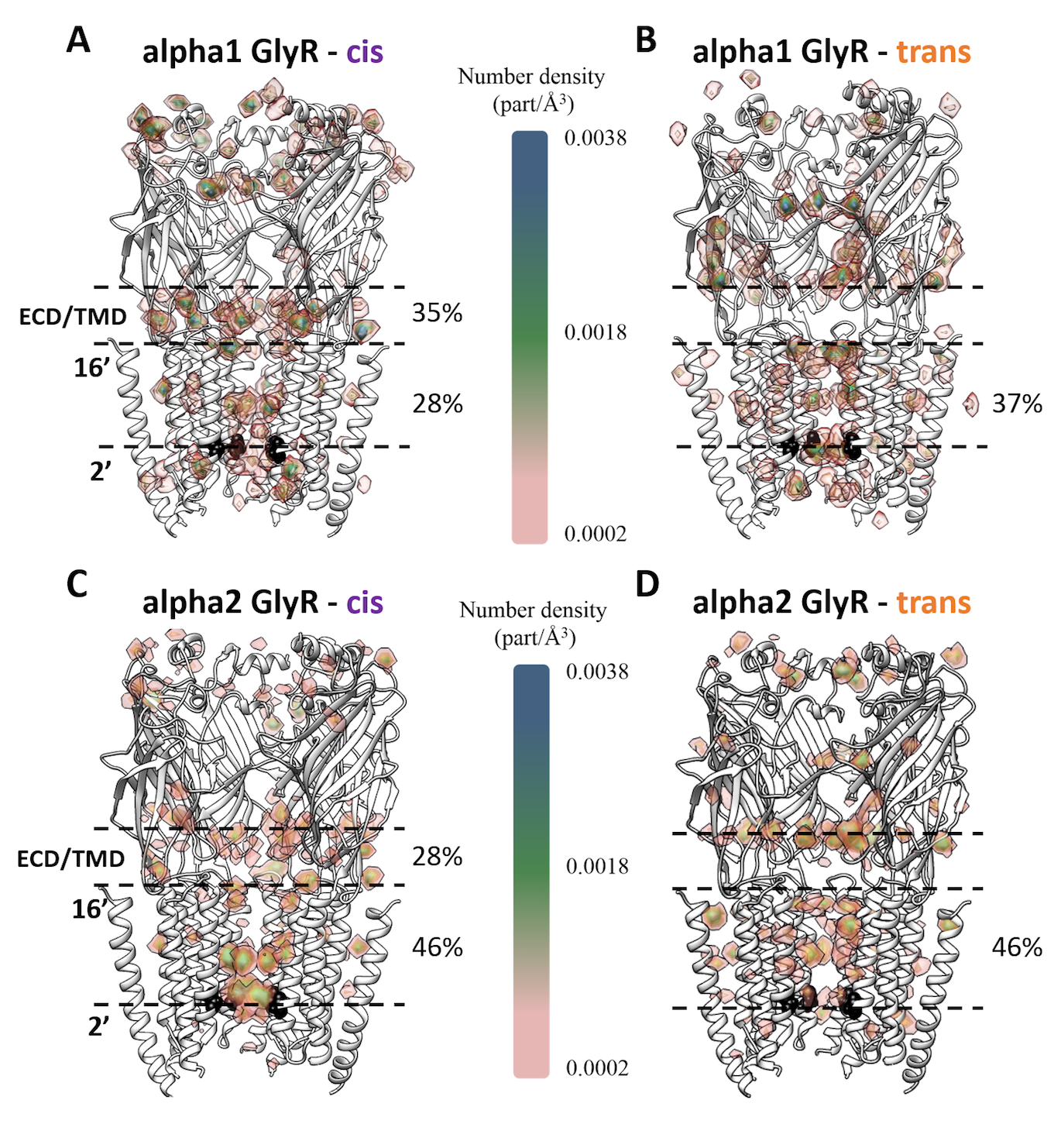

Supplement: Extended Data Figure 7-7 — Density map of the sulfonate group of Azo-NZ1 resulting from the blind docking to (A, B) α1 and (C, D) α2 GlyRs. Regions of continuous density correspond to higher sulfonate occupancy and indicate tighter sulfonate binding. Each contour line corresponds to 0.0006 particle/nm3, where “particle” refers to the sulfonate group in a given binding pose and is used to follow the putative interaction regions of Azo-NZ1. Longitudinal view of the receptor: the front subunit is not displayed for the sake of clarity. The pore region (between positions S/T16’ and G/A2’), as well as the interface region between the ECD-TMD, are marked with dashed lines. The residue at position 2’ is displayed as black spheres. Trans-Azo-NZ1 binds preferentially inside the 16’–2’ region for both GlyRs (37% and 46% for α1 and α2 GlyRs, respectively). Instead, cis-Azo-NZ1 shows two possible interaction sites, either inside the pore or at the ECD-TMD interface. The relative percentage of poses in the two regions varies significantly between the two GlyR types. For α1 GlyR, cis-Azo-NZ1 has a higher probability to interact with the ECD-TMD interface (35%) than with the pore (28%). In contrast, for α2 GlyR, the sulfonate density in the interface region is more dispersed, and the associated probability is lower (28%) than the one corresponding to binding in the pore (46%). Download Figure 7-7, TIF file. [file enu-eN-NWR-0294-20-s09.tif]
